# Investigation on the Synergy between Membrane Permeabilizing Amphiphilic α-Hydrazido Acids and Commonly Used Antibiotics against Drug-Resistant Bacteria

**DOI:** 10.3390/molecules29174078

**Published:** 2024-08-28

**Authors:** Cristina Minnelli, Gianmarco Mangiaterra, Emiliano Laudadio, Barbara Citterio, Samuele Rinaldi

**Affiliations:** 1 Department of Life and Environmental Sciences, Polytechnic University of Marche, Via Brecce Bianche, 60131 Ancona, Italy; c.minnelli@staff.univpm.it; 2 Department of Biomolecular Science, University of Urbino “Carlo Bo”, 61032 Urbino, Italy; gianmarco.mangiaterra@uniurb.it; 3 Department of Science and Engineering of Matter, Environment and Urban Planning, Polytechnic University of Marche, Via Brecce Bianche, 60131 Ancona, Italy; e.laudadio@staff.univpm.it

**Keywords:** α-hydrazido acid derivatives, membrane permeabilization, molecular dynamics, antibiotic adjuvants, antibiotic resistance, synergy

## Abstract

The growth of (multi)drug resistance in bacteria is among the most urgent global health issues. Monocationic amphiphilic α-hydrazido acid derivatives are structurally simple mimics of antimicrobial peptides (AMPs) with fewer drawbacks. Their mechanism of membrane permeabilization at subtoxic concentrations was found to begin with an initial electrostatic attraction of isolated amphiphile molecules to the phospholipid heads, followed by a rapid insertion of the apolar portions. As the accumulation into the bilayer proceeded, the membrane increased its fluidity and permeability without being subjected to major structural damage. After having ascertained that α-hydrazido acid amphiphiles do not interact with bacterial DNA, they were subjected to synergy evaluation for combinations with conventional antibiotics. Synergy was observed for combinations with tetracycline against sensitive *S. aureus* and *E. coli*, as well as with ciprofloxacin and colistin against resistant strains. Additivity with a remarkable recovery in activity of conventional antibiotics (from 2-fold to ≥32-fold) together with largely subtoxic concentrations of α-hydrazido acid derivatives was found for combinations with ciprofloxacin toward susceptible *S. aureus* and methicillin toward MRSa. However, no potentiation of conventional antibiotics was observed for combinations with linezolid and gentamicin against the corresponding resistant *S. aureus* and *E. coli* strains.

## 1. Introduction

The continuously accelerating spread of antimicrobial resistance (AMR), frequently in the form of multidrug or pan-resistance [1], has been recently worsened by the strong slowing down in the discovery of new antibiotics in recent decades and is now a major global health threat [2]. According to the World Health Organization (WHO), the annual global rate of deaths caused by MDR bacteria is nowadays around two million people, but it could reach about ten million people in 2050 [3,4].

In this alarming scenario, antimicrobial peptides (AMPs) have recently been considered as a potential new class of antibiotics [5]. They act mainly as membrane permeabilizing/disrupting agents, due to the interaction of their amphiphilic active structures with phospholipid bilayers, even if a few of them display intracellular targets [6]. AMPs are usually highly positively charged and thus they interact preferentially with negatively charged membranes, such as those of bacterial cells [7], but the selectivity toward mammalian cells is still reduced, so that their systemic administration has been limited so far to polymyxins, which are, however, quite toxic [8]. Other major issues limiting the development of AMPs for systemic use are their inherent susceptibility to enzymatic degradation and their high production costs [9], even though the possibility of a rational design with a reliable in silico approach can be attractive [10]. However, besides their undeniable drawbacks, AMPs have also demonstrated the potential to act synergistically with conventional antibiotics, thus paving the road to the use of surfactant-like compounds in combination with already existing antimicrobial drugs in order to fight AMR [11,12], exploiting an approach that showed good potential even when synergy was obtained by combinatorial use of antibiotics and repurposed nonantibiotic drugs [13].

To afford amphiphiles that are synthetic mimics of AMPs, different routes have been explored. The correct placement of lipophilic and cationic monomers in oligomeric sequences made of unnatural analogs of α-amino acids led to amphiphilic foldamers that act as antimicrobial peptidomimetics [14] and show interesting potential as membranolytic compounds, as mainly demonstrated for β-peptide oligomers [15], short peptoid structures [16], and also ureas [17]. However, albeit not being susceptible to proteases, foldamers need a multistep solid phase synthesis and are quite expensive; thus, they are not suitable for production on an industrial scale. Another successful strategy was the use of smaller molecules, whose amphipathicity was either due to the use of preorganized building blocks [18,19,20,21,22] or assumed only upon interaction with the target bilayer [23,24,25,26,27,28]. However, despite the continuous improvement in performances, both foldamers and smaller molecular amphiphiles have not yet reached a satisfying level of selectivity toward mammalian cells. Eventually, different polycationic polymers exhibited promising features, but even though many of them have been successfully developed as antimicrobial hydrogels and coatings, antibiofilm agents, immunomodulators and adjuvants for conventional antibiotics, they can hardly be employed by systemic administration [29].

Following the idea of synthesizing a simple structure in which the presence of noncovalent interactions could help in obtaining a preorganized amphiphilic structure, we devised that the intramolecular bifurcated hydrogen bond characterizing the peculiar secondary structure of foldamers obtained from short imidazolidinone-tethered α-hydrazidopeptides [30] might also be formed in properly derivatized monomers. To this end, a series of rationally designed monomeric α-hydrazido acid derivatives lacking the cyclic conformational constriction of their imidazolidinone-tethered counterparts was easily synthesized in five steps, using only cheap starting materials and obtaining excellent overall yields (32–72%) without the need for preparative HPLC in order to purify the free amines. After salification of the glycine side chains, the hydrochlorides were then submitted to evaluation of minimum inhibitory concentration (MIC), hemolytic concentration (HC_50_), stability toward enzymatic and chemical degradation, membrane permeabilization activity, and importance of both the global lipophilicity and the non-disrupted amphiphilicity [31].

**Figure 1:**
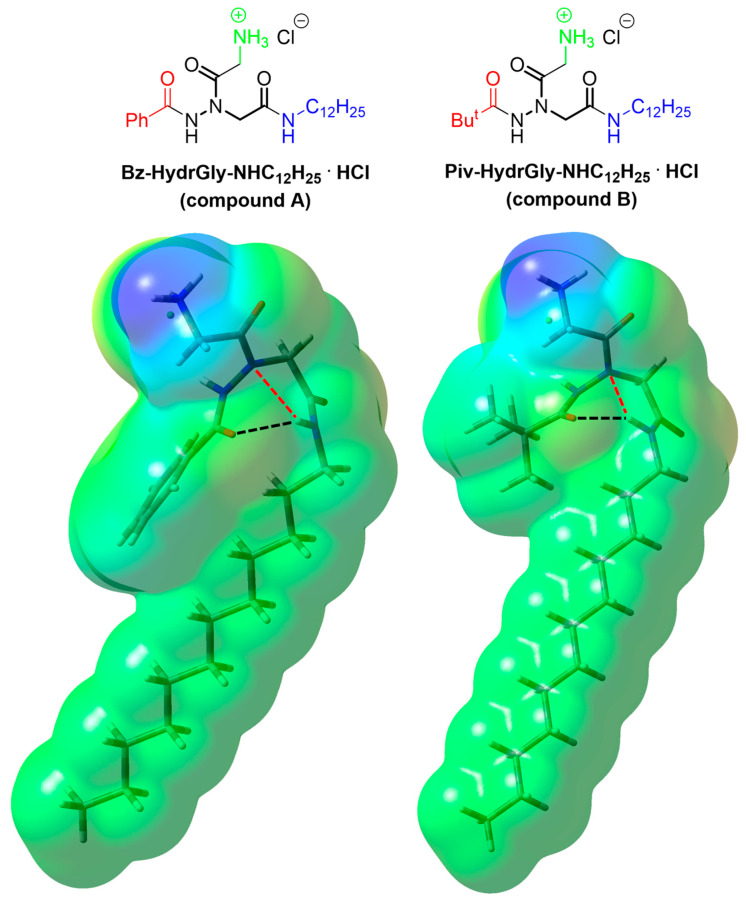
Amphiphilic α-hydrazido acid derivatives used in this work and tridimensional structures of their ammonium cations, for which the electrostatic potential surfaces are also reported. The α-hydrazido acid unit is in bold, while the N-terminal acyl, C-terminal amino and ammonium unit of glycine side chain are in red, blue, and green, respectively. Covalent + electrostatic hydrogen bonds (black dotted lines) and purely electrostatic hydrogen bonds (red dotted lines) are reported. The negatively charged chloride anions are on the rear side and their surfaces are only partly visible.

Briefly, when both the N- and C-terminal of the hydrazido acid moiety were properly substituted with an acyl and an amino group, respectively, the formation of a hydrazido-turn secondary structure with a bifurcated hydrogen bond ensured the correct segregation of lipophilic and hydrophilic portions in these monocharged amphiphiles, whose ammonium cation was obtained by tert-butoxycarbonyl deprotection of the glycine side chain. Among the many different compounds tested, derivatives **A** and **B** in Figure 1 evidenced the best global values in terms of antibacterial and hemolytic activities, showing therapeutic indices that, albeit still not sufficient for practical implementations, were among the highest in the literature for amphiphilic membranolytic compounds.

Compounds **A** and **B** were active against different bacteria, comprising either collection or multidrug-resistant (MDR) strains of both Gram-positive and Gram-negative bacteria, albeit *P. aeruginosa* was slightly less susceptible. At concentrations equal to the corresponding MICs, the compounds showed a rapid permeabilizing action on bacterial membrane(s), even on the MDR *E. coli* 288328 strain with resistance to colistin, which was caused by the addition of phosphoethanolamine to the otherwise negatively charged lipid A of lipopolysaccharide (LPS), leading to a largely decreased interaction with the many ammonium groups of colistin [32]. The hemolytic concentration, which is commonly used as the primary indicator of the minimal required type of selectivity for membranolytic compounds against mammalian cells, showed HC_50_ values of 195 ± 13 and 394 ± 28 μg/mL for compounds **A** and **B**, respectively. Even though they are not still enough, they are among the best values found in the literature for compounds acting on membranes. In addition, amphiphilic α-hydrazido acids were demonstrated to be perfectly stable against chemical degradation for prolonged times as refrigerated solid hydrochlorides, but also stable in aqueous solutions, at least in a non-basic medium, and they were resistant toward enzymatic degradation. This feature gives these compounds an advantage over the proteolytically sensitive α-peptidic AMPs, which are also more expensive, while their ease of synthesis at low cost makes them economically advantageous in comparison to their oligomeric counterparts (i.e., foldamers). Eventually, the importance of the overall lipophilicity and the amphiphilic balance for the selectivity with respect to mammalian cells, as well as the role of non-disrupted amphiphilicity in antimicrobial activity, were assessed [31].

Since these compounds showed the ability to permeabilize the bacterial membrane(s) of both Gram-positive and Gram-negative bacteria, we envisaged that amphiphilic α-hydrazido acids could act with an additive effect, or even synergistically, in combination with conventional antibiotics. Thus, herein we report the results of experiments conducted with various couples α-hydrazido acid/conventional antibiotic, using bacterial strains that are either susceptible or not susceptible to the conventional antibiotic. The observed experimental results have been interpreted in view of (i) the action of amphiphilic α-hydrazido acids, (ii) the mechanism of resistance to the conventional antibiotic, (iii) the amount of resistance showed by the resistant strain in comparison to the corresponding susceptible strain, (iv) the potential effect in increasing the cytoplasmic concentration for conventional antibiotics having an intracellular target, and (v) the possible combined effect in attacking the same target for conventional antibiotics also acting on the membrane.

## 2. Results and Discussion

With the aim of drawing safer conclusions about the most important data, namely synergies or additivities deduced from chequerboard experiments (Section 2.6), we had to work out some preliminary details. First, we assessed whether, in the present experimental conditions, α-hydrazido acids interact with bacteria as micelles or as monomers (Section 2.1), because the presence of micelles could lead to both direct interactions with the common antibiotics used in combination (e.g., their encapsulation with a concomitant additional mechanism of uptake) and also possibly give a more complex mechanism of action onto membranes, different from a generic membrane permeabilization. Then, the ability of α-hydrazido acids to permeabilize the bacterial membrane(s) even at subtoxic concentrations was examined (Section 2.2), establishing whether or not they could potentially be able to increase the uptake of common antibiotics at the adjuvant concentrations that are relevant for synergies and additivities. The following set of molecular dynamics (MD) simulations (Section 2.3) was used to theoretically confirm the previous findings about the membrane-permeabilizing action of compounds **A** and **B**, and also to add important details about the reasons at the basis of the experimentally observed effects. To better discuss the results of combinations of common drugs and α-hydrazido acids against Gram-negative bacteria, the binding of **A** and **B** with LPS was evaluated in Section 2.4. Eventually, a further potential mechanism of action that characterizes some AMPs (i.e., inhibition of DNA replication) was excluded for the present amphiphilic compounds, showing that they act purely as membrane permeabilizers (Section 2.5).

### 2.1. Determination of Critical Micellar Concentrations (CMC)

**Figure 2:**
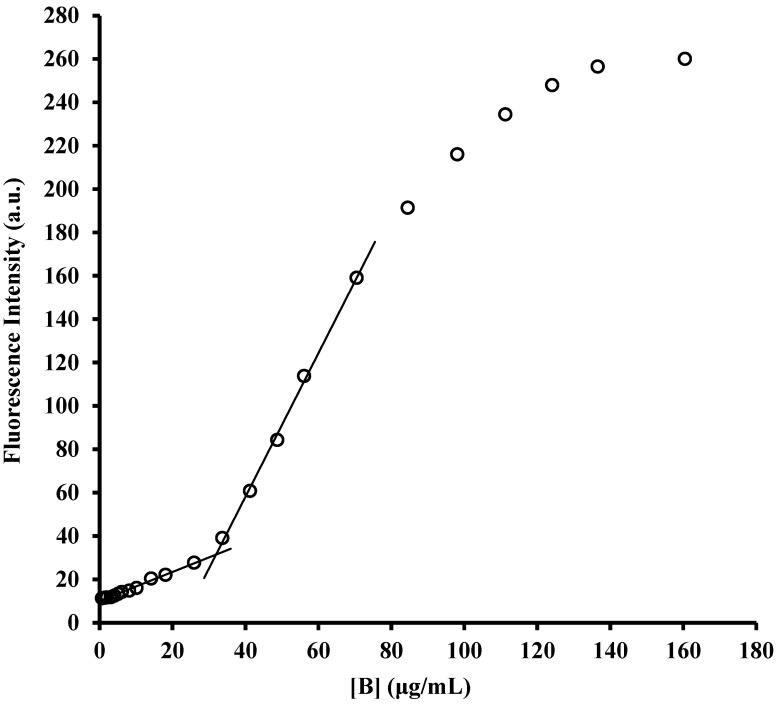
Determination of critical micellar concentrations by fluorimetric titration. Representative curve of fluorescence as a function of concentration of compound **B** added to a 1 μM solution of the fluorescent probe *N*-phenyl-1-naphthylamine (NPN) in 150 mM phosphate buffer solutions (PBS, pH 7.4). CMC was calculated as the intersection point between the two lines traced before and after the increase in fluorescence. CMCs reported in the text are the average results of three tests for each compound.

The possibility of forming aggregates that do not act by a generic permeabilization or depolarization of bacterial membrane(s), but instead by causing major damage that eventually leads to its (their) complete collapse, has been demonstrated to be a possible mechanism for cationic oligomeric surfactants. Indeed, sequential disruption of the outer and then the inner membrane was accomplished by those aggregates in the case of *E. coli* [33]. To investigate the putative synergistic activity with conventional antibiotics, it was important to evaluate if our amphiphiles could also act with this mechanism especially at subtoxic concentrations. To this end, their critical micellar concentrations (CMCs) were measured under physiological conditions (PBS, pH 7.4) (Figure 2), the same used for the evaluation of MIC and membrane permeabilization. In addition, the use of PBS instead of water has been shown to be mandatory to correctly interpret data for the cationic oligomeric surfactants, because in pure water the lack of added salts increases the electrostatic repulsion between ammonium groups and also possibly diminishes the hydrophobic effect, due to extremely reduced ionic strength, thus leading to CMCs in water that are about two orders of magnitude larger than those in PBS [33].

The CMCs obtained by fluorimetric titration of *N*-phenyl-1-naphthylamine (NPN) in PBS were 13 µg/mL for compound **A** and 32 µg/mL for compound **B**. Therefore, comparing these data with the highest MICs measured for **A** and **B** against the different bacterial strains tested (8 µg/mL for **A** and 16 µg/mL for **B**), it can be safely deduced that our compounds do not act as micelles or other aggregates when they are used alone, but they are instead simply membrane(s) permeabilizers, and this is even more certain at the subtoxic concentrations adopted in combination with the antibiotics in synergy assays. This also confirms that the interactions between α-hydrazido acids and antibiotics only occur at a mechanistic level and rules out the possibility of a direct interaction at the molecular level, such as an encapsulation of drugs in micelles of **A** and **B**, which could offer a different uptake mechanism when discussing synergies and additivities in Section 2.6.

**Figure 3:**
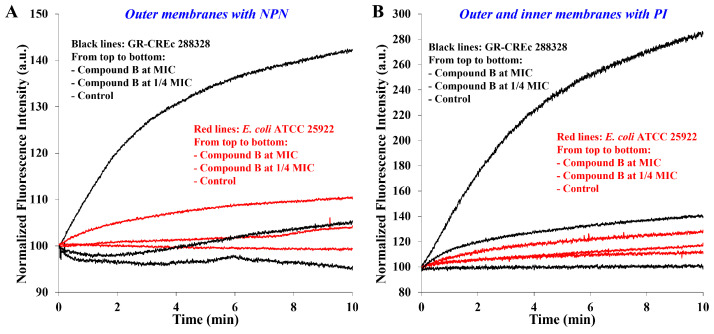
Variation of normalized fluorescence intensity with time caused by different concentrations of compound **B** [control (compound **B** not added), ¼ MIC, MIC] for (**A**) permeabilization of outer membranes (fluorescent probe: NPN) and (**B**) permeabilization of both outer and inner membranes (fluorescent probe: PI) in the *E. coli* ATCC 25922 collection strain (red lines) and in the gentamicin and colistin resistant GR-CREc strain (*E. coli* 288328, black lines). Data for control and compound **B** at MIC taken from Ref. [31].

These findings reinforce the results reported in the next section for the permeabilization of outer and inner membranes of *E. coli* ATCC 25922 and multidrug-resistant GR-CREc *E. coli* 288328 at MIC and ¼ MIC (Figure 3), which did not show any abrupt change in fluorescence, as would be expected in the case of a complete collapse of bacterial wall.

### 2.2. Membrane Permeabilization at Subtoxic Concentrations of α-Hydrazido Acids

In the previous work, it was already demonstrated that the main mechanism of action of α-hydrazido acids is the permeabilization/destabilization of bacterial phospholipid bilayer(s) [31]. To this end, compound **B** was used as a model system against a collection (ATCC 25922) and a gentamicin and colistin resistant (GR-CREc, 288328) *E. coli* strain. Although only the most evident results obtained at concentrations of compound **B** equal to its MICs were previously reported, experiments at largely subtoxic concentrations (i.e., ¼ MIC) were also conducted, and their results were added in Figure 3 to the original sets of data. NPN indicates the outer membrane (O.M.) permeabilization by augmenting its fluorescence when passing from an aqueous solution to the outer membrane, while propidium iodide (PI) indicates the permeabilization of both membranes by binding to nucleic acids.

The experimental findings previously reported that are relevant in the present case can be briefly summarized as follows. Without compound **B**, either NPN (Figure 3A) or PI (Figure 3B) showed no uptake for the GR-CREc strain, demonstrating that its lipopolysaccharide modified by addition of phosphoethanolamine [34] (i.e., more cationic) acts as an effective barrier for both fluorophores, while the slow non-promoted uptake of PI by the collection strain indicates that the standard LPS in *E. coli* ATCC 25922 is somewhat more permeable. This non-promoted uptake of PI is more evident when data in Figure 3B are plotted using a different normalization procedure, as reported in Figure S1 in Supplementary Material. Moreover, the time-dependent increase in membrane permeability is clearly due to the accumulation of amphiphile **B** into the phospholipid membrane(s). In addition, even though the two strains seemed to have dramatically different rates of uptake for both fluorescent probes in the presence of compound **B**, this was simply a visual effect due to large differences in terms of absolute fluorescence intensity. In fact, the computed rate constants for the exponential rise to maximum at a concentration of compound **B** equal to MIC were quite similar for both strains, the GR-CREc interestingly being slightly more susceptible. Eventually, the most important experimental observation was the powerful permeabilization of both membranes that occurred rapidly when the α-hydrazido acid hydrochloride **B** was present at its MIC value [31].

Permeabilization data from experiments with compound **B** at the subtoxic concentration of ¼ MIC (i.e., the middle red and black lines in both graphs in Figure 3) gave some important clues. First, even for such a short experimental time, 10 min, the permeabilizing effect was still evident, albeit fairly reduced in comparison to that obtained at MIC. For the *E. coli* ATCC 25922 collection strain, the much smaller changes in terms of absolute fluorescence reported above lead to initial portions of change in relative fluorescence that are almost superimposed with the control line for the permeabilization of both the outer (graph A) and the inner membrane (graph B). In particular, the normalized fluorescence intensity with ¼ MIC of compound **B** added diverges from the control line just before 2 min in the case of the outer membrane, whereas it takes almost 6 min for the inner membrane (I.M.). This observation seems to clearly indicate that, in the case of *E. coli* ATCC 25922 collection strain, there is the need for an initial accumulation of amphiphilic compound into the bilayer in order to reach the ability to permeabilize it at such a reduced concentration.

On the other hand, in the case of gentamicin and colistin-resistant GR-CREc *E. coli* 288328 strain, the experimentally much larger changes in absolute fluorescence and the slightly higher susceptibility to permeabilization already demonstrated with α-hydrazido acid **B** at MIC also apply at the subtoxic concentration (¼ MIC), making the divergence from the control line more visible. Nevertheless, also in this latter case it is evident, especially from Figure 3A, that there is a net increase in the permeabilization rate of outer membrane after about 2–3 min. In addition, even for the inner membrane (Figure 3B), despite the resemblance to an exponential rise to maximum and the quite good coefficient of determination (r^2^ = 0.988), the observed signal at ¼ MIC of compound **B** does not actually seem to be safely attributable to such a simple kinetic scheme after the visual comparison between its regression curve (Figure S2 in Supplementary Material) and the one obtained at MIC, which gave instead an almost perfect nonlinear regression (r^2^ = 0.999, Figure S3).

Another interesting observation can be made considering the previously computed values for the permeabilization of O.M. alone (Figure 3A) and both the O.M. and I.M. consecutively (Figure 3B) [31]. The values measured for both GR-CREc (0.31 min^−1^ for the O.M., 0.24 min^−1^ for O.M + I.M.) and *E. coli* ATCC 25922 (0.25 min^−1^ for the O.M., 0.22 min^−1^ for O.M. + I.M.) strongly confirm that the outer membrane is by far harder to permeabilize than the inner one. In fact, the overall constant (i.e., O.M. + I.M.) is in both cases quite close to the value for the outer membrane alone, and this is especially true for *E. coli* ATCC 25922 strain. Thus, even without experiments or in-depth computational investigations aimed at precisely defining the behavior of compounds **A** and **B** with different membranes [35,36], we could safely ascertain that the main obstacle to the action of our α-hydrazido acids against Gram-negative bacteria is the outer membrane, and this will be important when dealing with synergies in Section 2.6.

The final observation about permeabilization data is the fact that, as expected, the effect observed for subtoxic concentrations is much slower than that obtained at MICs, and this will be taken into proper consideration when analyzing the results of chequerboard assays in Section 2.6.

### 2.3. Molecular Dynamics Simulations

**Figure 4:**
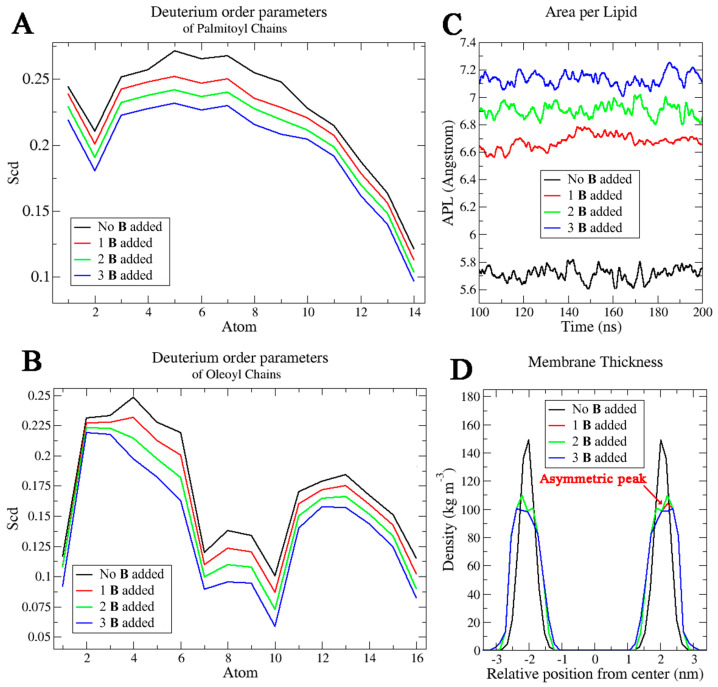
Computational analysis of membrane properties as a function of the number of molecules of compound **B** added (black lines: without compound **B**; red lines: one molecule of **B**; green lines: two molecules of **B**; blue lines: three molecules of **B**). (**A**) Deuterium order parameter (Scd) of palmitoyl chains, (**B**) deuterium order parameter (Scd) of oleoyl chains, (**C**) area per lipid, and (**D**) membrane thickness.

To clarify the mechanism of interaction with the membranes, MD simulations were performed considering compounds **A** and **B** and a solvated POPE-POPG mixed lipid system, which mimics the composition of the typical inner membrane of *E. coli*, and is usually taken as a reference in this kind of calculations [18], whereas considering other more specific systems (i.e., outer membranes of Gram-negative bacteria, containing LPS, or membranes of Gram-positive bacteria) was out of the scope of these simulations. To consider even the smallest influences of amphiphilic compounds **A** and **B** on the membrane, different membrane properties such as deuterium order parameters for oleic and palmitic acids, area per lipid (APL), and membrane thickness were calculated through MD trajectories in the pure lipid system and in the systems with one, two and three molecules of amphiphilic α-hydrazido acids. This approach was chosen in order to verify the effect of increasing the concentration of α-hydrazido acids into the POPE-POPG bilayer, without focusing on a particular number of amphiphilic compound molecules into it, because the exact value is unknown and is also strongly time-dependent, as experimentally ascertained by the permeabilization assays (Figure 3). The results reported here are for compound **B** (Figure 4), whose corresponding experimental membrane permeabilizations are reported above, but almost identical data were obtained for compound **A** (see Supplementary Figure S4). While for the APL the whole MD trajectories were considered, the deuterium order parameter and the membrane thickness were calculated considering the last 40 ns of MD simulations, to discard the initial pathway in which the systems did not yet reach the steady state. This is crucial because, even if it is known that this type of molecules interacts effectively with the charged polar portions of membranes, also the α-hydrazido acid C-terminal aliphatic chain and the N-terminal aromatic moiety certainly play an important role in the membrane permeabilization effect, since the polar interactions constitute only the first step of molecule internalization in the bilayer.

The deuterium order parameters (Figure 4A) describe the motional disorder of the hydrocarbon chains. Atoms toward the chain terminus have lower values, indicating that these atoms have no preferential orientation and that they can fluctuate more than the other carbon atoms of the chains. Comparing the systems with and without compound **B**, the presence of a single α-hydrazido acid molecule decreased the order degree of both chain types. This suggests an increased permeabilization which is attributable to an increased fluidity of the membrane. In particular, the most evident decrease was detected for the upper part of the chains, and this is an indication about the preferential displacement of the molecule in this region of the bilayer. This trend was more evident increasing the number of compound **B** molecules in simulations, with a gradual reduction of the order degrees for both the chain types (Figure 4A,B).

APL values (Figure 4C) calculated along the MD simulations showed an average value of 5.73 ± 0.09 Å^2^ for the system without any molecule of amphiphilic compound, and this was maintained for the whole MD time. A peculiar trend was observed when compound **B** was included, since the APL values were the same as the previous system at the beginning of simulation, then increased after 10 ns of simulations to reach 6.63 ± 0.09 Å^2^, 6.86 ± 0.09 Å^2^, and 7.11 ± 0.09 Å^2^ for the systems with 1, 2, and 3 amphiphilic molecules of **B** added, respectively. Extremely similar values were obtained for the systems with 1, 2, and 3 amphiphilic molecules of compound **A** (i.e., 6.64 ± 0.08 Å^2^, 6.88 ± 0.09 Å^2^, and 7.15 ± 0.10 Å^2^, respectively). Based on experimental measurements of membrane structural properties, the permeability was shown to be strongly correlated with the APL [37]. This means that this important increase in APL values, which is extremely similar for compounds **A** and **B**, is a clear confirmation of the permeabilizing effect of these α-hydrazido acids on the membrane.

Finally, the membrane thickness was calculated focusing on the last 40 ns of MD simulations (Figure 4D). This parameter has been extracted by monitoring the relative position of the phosphate groups of the lipids as a function of the center of the membrane. The analysis showed a symmetrized representation of the thickness for the membrane without amphiphilic compounds, with a value of 4.2 ± 0.2 nm. When one amphiphilic α-hydrazido acid molecule was included, the membrane thickness assumed a slightly asymmetric plot, and this is correlated to the presence of the α-hydrazido acid in one of the two leaflets. Moreover, a small decrease in values was observed, reaching 4.02 ± 0.25 nm for compound **A** and 4.01 ± 0.25 nm for compound **B**. Increasing the number of amphiphilic molecules, the thickness remained the same, but the relative position of the phosphate groups appeared to be more perturbed. To conclude, with either compounds **A** or **B** inserted into the phospholipid bilayer, lipid membranes show decreased thickness and lower chain order, with an increased membrane area. As a result, the membranes have higher permeability for small solutes [38,39].

**Figure 5:**
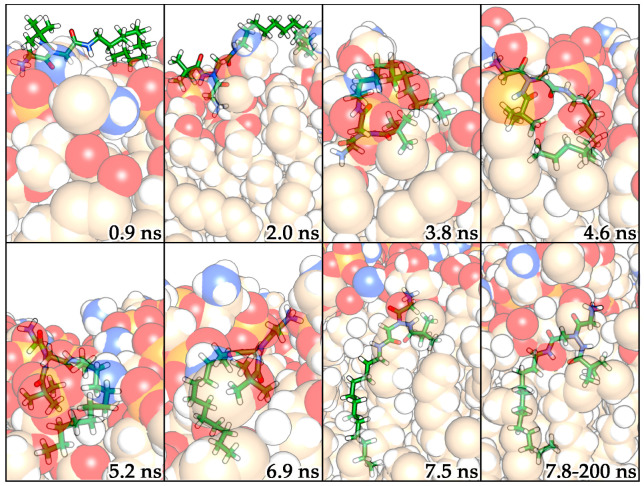
MD snapshots of α-hydrazido acid **B** (stick) and membrane (van der Waals sphere). O, N, P, and H atoms are highlighted in red, blue, orange, and white, respectively. The C atoms of compound **B** are reported in green, while those of POPG and POPE are reported in light brown.

The comparison between the systems with one, two, and three molecules of compounds **A** and **B** highlighted how the amphiphilic molecules tend not to interact with each other but, on the contrary, they are able to diffuse laterally in the lipid medium causing alterations in the membrane parameters. From the order parameters and thickness calculations, it emerged that the polar portion of the amphiphilic α-hydrazido acids strictly interacts with the hydrophilic component of the membranes, while their C12 chain extends towards the lipophilic core of the bilayer. This result indicates that the molecules do not undergo flip-flop phenomena moving from one leaflet to another in the time considered, but on the contrary they exhibit high lateral diffusion with a perturbative effect on the entire layer. To remark this behavior and to confirm the trend observed in the membrane parameters investigation, the mean-square displacement (MSD) of lipids was also calculated. The diffusion constants derived from the mean-square displacement at the end of MD simulations were 4, 5.2, and 5.7 nm^2^ s^−1^ for the systems with one, two, and three molecules of compound **B**, respectively, with compound **A** showing an almost identical behavior (Figure S5). These values are much higher than that obtained in the normal bilayer environment (3.5 nm^2^ s^−1^). Since, as mentioned, there are no flip-flop movements, the increase in MSD values is notable, further underlining the permeabilizing effect of the compounds on the membranes. To better understand the interaction dynamics of these molecules with lipids and their internalization into the bilayer, the MD trajectories were analyzed and reported in Figure 5 for compound **B**, while the corresponding snapshots for compound **A**, indicating an identical behavior, are in Figure S6.

After the equilibration phase, the molecule of α-hydrazido acid **B** quickly moved towards the polar portion of the membrane, making ionic interactions among the phosphate groups of lipids and the ammonium fragment of **B**. In this step, the aliphatic chain fluctuates on the lipid membrane together with the *tert*-butyl group (at 0.9 ns in Figure 5). At 2.0 ns, compound **B** orients its ammonium group deeper in the membrane, displacing the terminal portion of the aliphatic chain towards the membrane, and gradually begins to enter, while the *tert*-butyl group remains on the membrane surface. This process is relatively fast, as it is completed in approximately 3 ns (from 2.0 to 4.6 ns). During this short time, an interaction between the *tert*-butyl group and the terminal part of aliphatic chain of **B** is detected, then, after 5.2 ns, the chain gradually extends itself among the lipids as if it was a filament. After 7.5 ns, the molecule maintains the aliphatic chain in the lipid medium with a high movement freedom, while remaining anchored to the polar part of the membrane through its ammonium group. Moreover, the intramolecular hydrogen bond is maintained throughout the time in the membrane, despite the atoms’ fluctuations along MD simulations. This implies a relatively rigid orientation of the *tert*-butyl group, which remains displaced towards the upper part of aliphatic chains of membrane (Figure 5).

**Figure 6:**
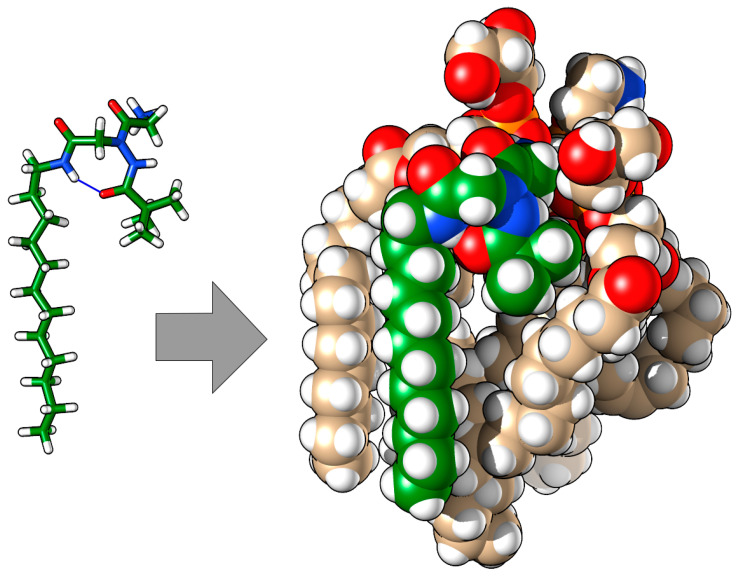
Representative structure of compound **B** in membrane. O, N, P, and H atoms are highlighted in red, blue, orange, and white, respectively. The C atoms of compound **B** are reported in green, while those of lipids are reported in light brown. The blue line between the H and carbonyl O of compound **B** indicates the intramolecular H-bond.

As a matter of fact, the N-terminal pivaloyl group of compound **B** (or benzoyl group in compound **A**, see Figure S7 in Supplementary Material) is the effective perturbative factor for the membrane, because it is able to laterally displace the lipids, whereas the highly mobile C-terminal linear aliphatic chain does not seem to introduce additional perturbations on the adjacent lipid chains. In addition, the strong intramolecular hydrogen bond is an important factor in directing a rigid N-terminal substituent, the pivaloyl or benzoyl groups in the present case, so that it pushes more effectively toward the lipid chains (Figure 6). This theoretical finding is in agreement with the fact that compounds **A** and **B** resulted to be more potent antimicrobials in comparison to their analogues having about the same overall lipophilicity, but with an N-terminal linear aliphatic chain [31].

### 2.4. LPS Binding Assay

**Figure 7:**
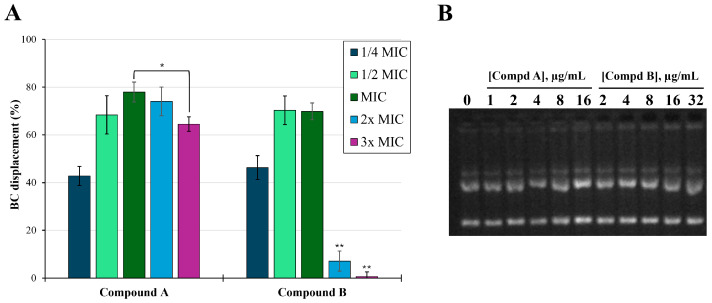
(**A**) BODIPY-TR cadaverine (BC) fluorescent displacement assay. Appropriate aliquots of α-hydrazido acids, referring to the ¼ MIC, ½ MIC, 2× MIC and 3× MIC of compounds **A** and **B** against *E. coli* ATCC 25922, were added to the aqueous solution of LPS:BC complex and fluorescence was recorded. Colistin solution (60 μg/mL) was used as reference for the complete disaggregation of LPS. * *p* < 0.05 and ** *p* < 0.01. (**B**) Gel retardation assay. Gel electrophoresis after addition of appropriate aliquots of compounds **A** and **B** and incubation with 100 ng of bacterial plasmid pBR322 in binding buffer for 1 h at 37 °C.

After investigating the behavior of amphiphilic α-hydrazido acids themselves in PBS solution (Section 2.1), and as generic membrane permeabilizers (both experimentally and theoretically, Section 2.2 and Section 2.3), for the main point of this work (i.e., the behavior when in combination with antibiotics), it was also important to have a better insight on the capability of **A** and **B** to bind not only generically to phospholipid membranes, but also specifically to LPS. LPS is the main pathogenic component of Gram-negative bacteria such as *E. coli* and *P. aeruginosa*, and is located at the outer membrane, creating the first barrier against antibiotics and bactericidal agents and contributing to the structural membrane integrity. Moreover, LPS has an endotoxin function and induces strong inflammation [40]. The binding of AMPs to LPS is known to be one of the main mechanisms by which they exert their anti-Gram-negative bacterial activity [41,42] and anti-inflammatory activity [43,44]. Therefore, to evaluate the ability of compounds **A** and **B** to selectively bind LPS, displacement assay of the fluorescent probe BODIPY-TR cadaverine (BC) was used. BC interacts specifically with the lipid A domain of the LPS, causing a self-quenching of fluorescence [45]. In Figure 7A, data obtained by addition of suitable aliquots of α-hydrazido acids to a solution of BC complexed with LPS from *E. coli* 0111:B4 are reported referring to the MICs of compounds **A** and **B** against *E. coli* ATCC 25922, which are 4 µg/mL for **A** and 8 µg/mL for **B** (Table 1), and using a 60 μg/mL colistin solution as reference for the complete disaggregation of LPS. Both amphiphilic hydrochlorides induced a dose-dependent increase in fluorescence intensity, underlining their ability to interact with LPS causing a BC displacement of about 40% already at ¼ MIC, and then increasing the fluorescence up to concentrations equal to their MICs. However, at concentrations equal to or higher than 2× MIC, **B** induced a very strong decrease in BC fluorescence, and a similar trend was also observed for compound **A**, albeit in this latter case the decrease in BC fluorescence (i.e., from 78% at MIC to 60% at 3× MIC) was much less dramatic. Very likely, this behavior can be correlated with the self-quenching of the initially displaced BC probe, caused by its accumulation into mixed micelles formed by amphiphilic compounds and LPS as the concentration of added α-hydrazido acid hydrochlorides raises. This phenomenon does not occur with colistin due to its different mechanism of action, mainly based on the displacement of divalent cations from the negatively charged phosphate groups of LPS [32].

### 2.5. DNA Binding Assay

Based on the previous results, amphiphilic α-hydrazido acids inhibit bacterial growth by permeabilizing cell membranes, regardless of their composition. Due to the occurrence of a few cases in which natural AMPs also showed an additional mechanism of action targeting DNA and RNA, as it occurred for buforin II [46] and melittin [29], we wanted to evaluate if this could also happen for compounds **A** and **B**, even though their simple structure does not suggest this possibility. In this context, the potential binding of peptides to *E. coli* plasmid DNA was determined as a shift in the DNA bands after incubation with different concentrations of either **A** or **B**, ranging from ¼× MIC to 4× MIC referred to antimicrobial activity against *E. coli* ATCC 25922. As shown in Figure 7B, no retardation of DNA was observed at any concentration, indicating that DNA binding did not occur, and the antimicrobial activity, as well as the synergy or additivity found at subtoxic concentrations in combinations with common antibiotics, could mainly be ascribed to the above reported mechanism.

### 2.6. In Vitro Antimicrobial Susceptibility Assays

#### 2.6.1. α-Hydrazido Acids Antimicrobial Activity

Since we intended to evaluate the synergistic effect of the two α-hydrazido acids in association with different drugs, we added a few bacterial strains to the panel previously adopted in the in vitro assays [31]. In particular, we investigated the compounds’ activity against bacterial efflux pumps. Active efflux is considered to be one of the first lines of defense for bacteria against antimicrobials. In *S. aureus*, the main frequently overexpressed efflux pump is represented by NorA, which is able to extrude many structurally unrelated antibiotics, especially in MRSA strains [47]. Therefore, in addition to previously evaluated sensitive (i.e., *S. aureus* ATCC 29213, *E. coli* ATCC 25922) and multidrug-resistant strains with different mechanisms of resistance (i.e., linezolid- and methicillin-resistant *S. aureus* AOUC-0915, LR-MRSa, and gentamicin- and colistin-resistant *E. coli* 288328, GR-CREc) [31], we included the ciprofloxacin-resistant *S. aureus* SA1199B, which is a known NorA efflux pump overproducer [48], and the corresponding wild type strain, the ciprofloxacin-susceptible *S. aureus* SA1199. As shown in Table 1, compounds **A** and **B** confirmed the good activities toward either the susceptible or resistant strains that had been already tested, and satisfactory MIC values were also obtained against both ciprofloxacin-susceptible and resistant strains *S. aureus* SA1199 and SA1199B, and this can be related to the permeabilizing action of our α-hydrazido acids.

**Table 1:** In vitro antibacterial activity of α-hydrazido acids toward drug-sensitive and drug-resistant strains.

MIC (μg/mL) ^a^						
	*S. aureus*				*E. coli*	
Compd	ATCC 29213	AOUC-0915 ^b^	SA1199 ^c^	SA1199B ^d^	ATCC 25922	288328 ^e^
**A**	4	4	8	8	4	4
**B**	4	4	16	16	8	8

^a^ Conservative estimate of at least three independent assays. ^b^ Linezolid- and methicillin-resistant *S. aureus* AOUC-0915 (LR-MRSa). ^c^ Ciprofloxacin-susceptible *S. aureus* SA1199. ^d^ Ciprofloxacin-resistant *S. aureus* SA1199B. ^e^ Gentamicin- and colistin-resistant *E. coli* 288328 (GR-CREc).

#### 2.6.2. Combinations with First-Line Antibiotics

Then, the behavior in combination with conventional antibiotics was determined by the checkerboard method, calculating the fractional inhibitory concentrations (FICs). The values of ΣFIC were interpreted as follows, according to Odds’ suggestions: ≤0.5, synergy; >0.5 and <4, additivity or indifference; ≥4, antagonism [49]. However, since different interpretations were proposed [50,51,52,53], to avoid confusion we considered ΣFIC ≤ 0.5 as synergy (light green cells in Table 2) and took into consideration the actual values when discussing cases with ΣFIC > 0.5. Antagonism was never observed.

The panel of (multi)drug-resistant bacteria and corresponding drugs was selected adopting bacterial targets that were mostly independent from that of α-hydrazido acids. Even in the case of colistin, acting on the bacterial outer membrane, the mechanisms of action of the drug and the amphiphilic α-hydrazido acids are different [54], so that a synergistic effect might also be observed, as it occurred for example with combinations of antimicrobial peptides and peptoids [55]. In addition, we wanted to ascertain if α-hydrazido acids could also be useful in combination with a first-line antibiotic against sensitive strains, so that reduced side effects might be expected due to the lower concentration of antibiotic. To this end, we selected tetracycline as a hydrophilic antibiotic with a slow influx through bacterial membrane lipids when in the protonated form at a pH under its pKa (7.7) [55].

The main findings, which will be discussed in detail below, can be summarized as follows: (i) synergistic combinations were found for amphiphiles with tetracycline against susceptible *S. aureus* and *E. coli* strains; (ii) additivity could be obtained with ciprofloxacin against sensitive *S. aureus*, whereas synergy was observed for compound **B** with the corresponding resistant strain; (iii) the combination **A**/methicillin against the MRSa gave additivity, but with an unexpected very pronounced resensitization to methicillin; (iv) strong synergy was observed for α-hydrazido acids and colistin toward the resistant *E. coli* strain; (v) linezolid and gentamicin showed pure additivity in combination with both hydrochlorides when tested against the resistant *S. aureus* and *E. coli* strains, respectively.

**Figure 8:**
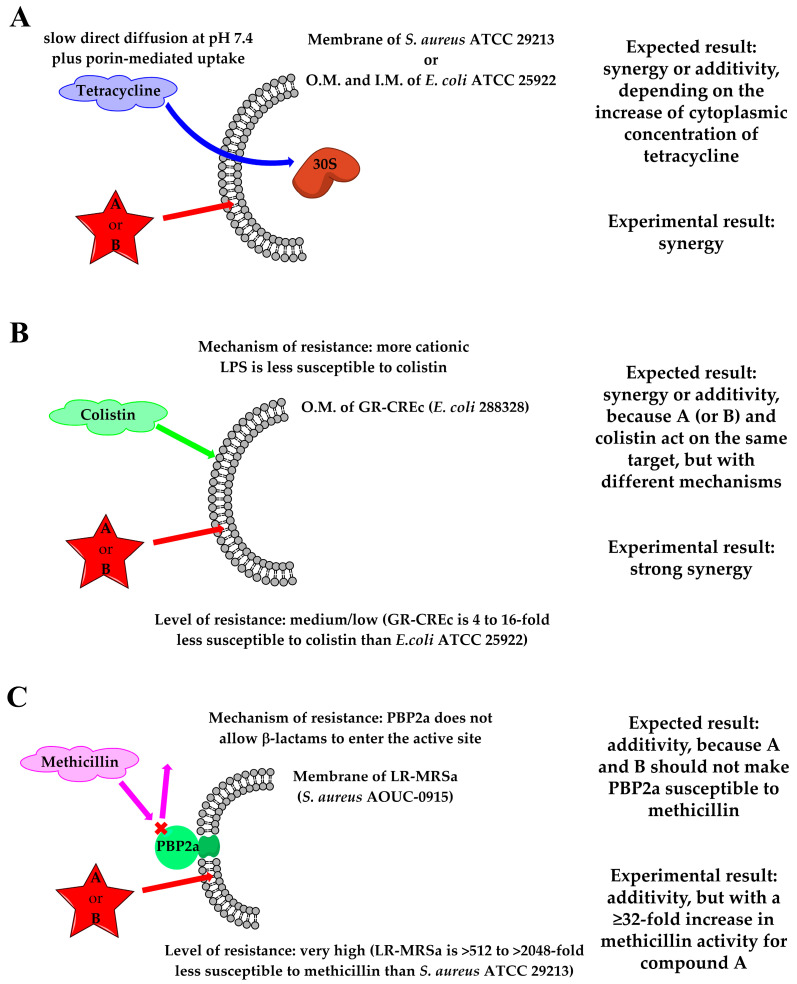
Schematic representations of the results observed for combinations of amphiphilic α-hydrazido acid derivatives **A** and **B** with (**A**) tetracycline against sensitive *S. aureus* ATCC 29213 and *E. coli* ATCC 25922, (**B**) colistin against GR-CREc, and (**C**) methicillin against LR-MRSa. Only the bacterial components relevant to the discussion are reported.

For combinations of **A** and **B** with tetracycline, at the experimental pH of 7.4, the presence of largely subtoxic concentrations of amphiphilic and permeabilizing compounds could substantially increase the influx of tetracycline and ultimately its concentration inside the bacterial cells. To our delight, both compounds **A** and **B** resulted in synergy with tetracycline against both sensitive *S. aureus* ATCC 29213 and *E. coli* ATCC 25922 strains (Table 2), suggesting a substantial increase in cytoplasmic concentrations of tetracycline that could reach its target (i.e., the small 30S ribosomal subunit, Figure 8A) [56].

Moreover, due to the fact that interpolated percent hemolysis at concentrations equal to their MICs was already demonstrated to be very reduced for compounds **A** and **B** in the absence of tetracycline [31], this synergy-based substantial reduction in the amounts of amphiphilic compounds needed would lead to a substantially null hemolysis.

Studying the combinations of the two amphiphilic α-hydrazido acids, **A** and **B**, with first-line antibiotics against drug- and multidrug-resistant (MDR) bacteria, different isolates were used. The linezolid- and methicillin-resistant *S. aureus* AOUC-0915 (LR-MRSa) carries both the mecA gene, responsible for methicillin resistance, and the cfr gene, responsible for linezolid resistance [57]. The mutant/susceptible strain pair SA1199B/SA1199 are both methicillin-susceptible, but the SA1199B isolate shows additional mutations that affect the quinolone resistance-determining regions (QRDR) of DNA gyrase and topoisomerase IV [58,59], which therefore have much less affinity for quinolones and fluoroquinolones, and also lead to the overexpression of the NorA efflux pump [48], thus conferring two different resistance mechanisms to ciprofloxacin. The gentamicin- and colistin-resistant *E. coli* 288328 (GR-CREc) possesses the *mcr-1* gene, responsible for colistin resistance [60], and the *aac(3)-IIa* gene, responsible for gentamicin resistance [58].

These results of checkerboard assays were interpreted, case by case, by considering the uptake, the mechanism of action of the different antibiotics, the cause and the extent of the bacterial resistance to a particular antibiotic in any of the evaluated strains, and the permeabilizing effect of α-hydrazido acids.

Both compounds showed synergism with colistin against GR-CREc 288328. Colistin activity mainly consists of the displacement of divalent cations and then the destabilization of lipopolysaccharide (LPS), the main component of the Gram-negative outer membrane, leading to leakage of the cytoplasmic content and ultimately causing cell death [32]. The MCR-1 protein leads to addition of phosphoethanolamine to lipid A. Consequently, the binding between the more cationic LPS and colistin is slightly less effective [61], as witnessed by the quite restrained change of colistin MIC from the accepted range (0.5–2 μg/mL) for sensitive *E. coli* ATCC 25922 strain, to 8 μg/mL for GR-CREc 288328. In our study, compounds **A** and **B** had a powerful synergistic interaction with colistin against GR-CREc, leading to MICs of colistin in combination of 0.25–0.5 μg/mL (Table 2). Most likely, the residual outer membrane destabilizing action of colistin, together with the concomitant destabilization of underlying phospholipid bilayers elicited by amphiphilic α-hydrazido acids, still can promote a combined and synergistic potent disrupting action on membranes (Figure 8B). Similar synergisms have already been reported for colistin/AMPs combinations [62,63].

Methicillin, as the other β-lactams, disrupts cell-wall synthesis by inhibiting transpeptidase activity of penicillin-binding proteins (PBPs). Thus, even though the action of β-lactams on cell wall synthesis and the one of amphiphilic compounds damaging the phospholipidic membranes are both at the bacterial surface, they are actually separated by a mechanistic standpoint, and synergy is rarely observed for many different β-lactams/AMPs combinations [64,65,66,67], the few exceptions almost always regarding Gram-negative bacteria [68,69,70] and only very rarely Gram-positive ones [71]. However, those results have not been rationalized and they appear to be highly variable, depending on the particular AMP/antibiotic/bacterium combination. In the present case, the *mecA* gene encodes PBP2a, a transpeptidase with extremely low affinity for all β-lactams, except for last-generation cephalosporins [72]. In fact, the MIC of methicillin alone dramatically rises from 0.5–2 μg/mL for *S. aureus* ATCC 29213 [55] to >1024 μg/mL for LR-MRSa. Even if the computed intervals for the possible actual values of ΣFICs (0.5 < ΣFIC ≤ 0.53 for **A** and 0.5 < ΣFIC ≤ 1.0 for **B**) are out of the commonly accepted range for synergistic effects (Table 2), the ≥32-fold increase in methicillin activity (MIC = 64 μg/mL) in combination with 2 μg/mL of hydrochloride **A** must be emphasized. This remarkable increment in methicillin activity when in combination with compound **A**, and to a much more reduced extent with compound **B**, is not safely explainable. Since PBP2a is stably localized in the cell membrane, an increase in cytoplasmic methicillin concentration cannot be invoked in this case. Possible reasons based on the action of α-hydrazido acids on membrane phospholipids and/or directly on PBP2a can be putatively adduced (Figure 8C). The lack of affinity of PBP2a for β-lactams, but not to the recent 5th generation cephalosporins ceftaroline and ceftobiprole, led to the discovery that older β-lactams do not acylate serine S403 in the active site of PBP2a, which is completely inaccessible. On the contrary, ceftaroline and ceftobiprole can access it by allosteric regulation, the presence of ammonium functionalities in these drugs being essential to bind to allosteric site [73]. Possible conformational variations of PBP2a induced by environmental changes of lipid disposition around the protein, associated to the action of amphiphilic α-hydrazido acids on bilayers, could be involved in the observed increase in methicillin activity. However, if they were the only cause, a comparable effect should be detected for compounds **A** and **B**, which had always previously demonstrated very similar behavior. In our opinion, the striking difference between the capabilities of the two α-hydrazido acid hydrochlorides to promote a partial recovery in methicillin activity might be explained with a mechanism involving a more specific interaction with either the allosteric site or the salt bridges network of PBP2a. Likely, some π-stacking or cation-π interaction of the phenyl N-terminal group in **A** with one or more residues of PBP2a, which would be stronger than the simple van der Waals interactions of the aliphatic and almost spherical tert-butyl fragment in **B**, could act as an allosteric regulation and be at the basis of the experimental results.

**Figure 9:**
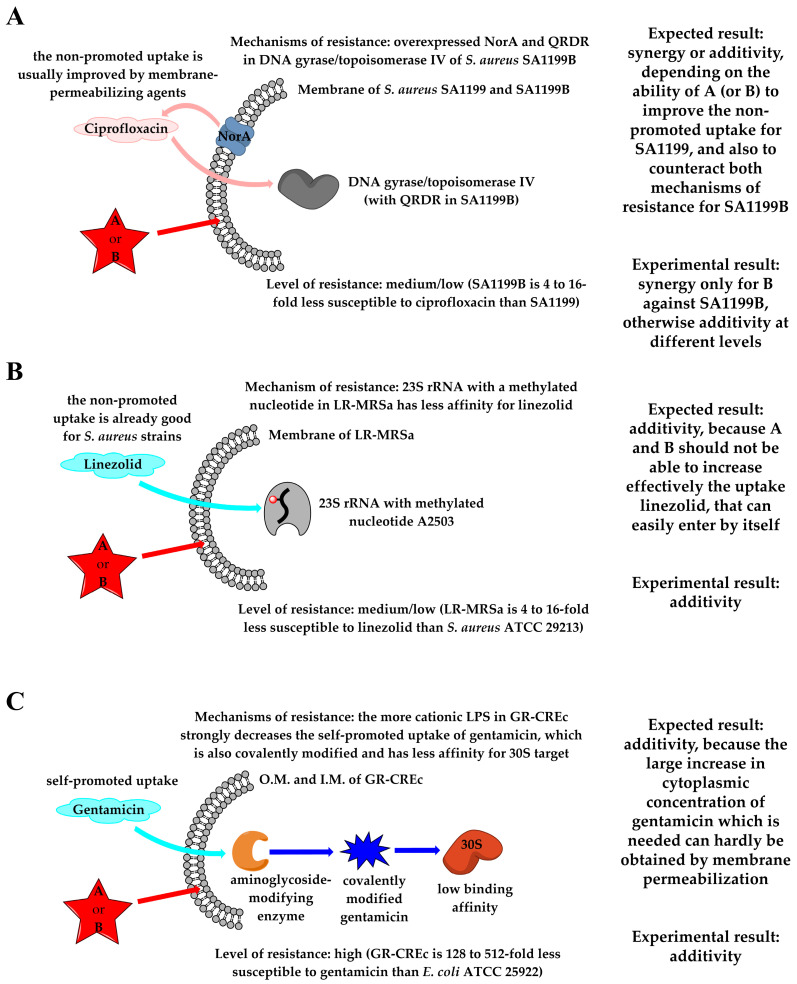
Schematic representations of the results observed for combinations of amphiphilic α-hydrazido acid derivatives **A** and **B** with (**A**) ciprofloxacin against sensitive *S. aureus* SA1199 and resistant SA1199B, (**B**) linezolid against LR-MRSa, and (**C**) gentamicin against GR-CREc. Only the bacterial components relevant to the discussion are reported.

The adjuvant effect observed in combination with ciprofloxacin for both compounds **A** and **B** can be considered as the consequence of the increased antibiotic uptake upon the bacterial membrane alteration (Table 2). It is remarkable that compound **B**, although reporting a two-fold higher MIC than compound **A** when used alone against both SA1199 and SA1199B (16 vs. 8 μg/mL, Table 1), was able to act synergistically with ciprofloxacin against the resistant strain (ΣFIC = 0.5) and to enhance the drug effectiveness against SA1199 (ΣFIC = 0.75), with a two-fold decrease in the MIC of ciprofloxacin in combination. Indeed, **B** was able to compensate both antibiotic efflux and the reduced affinity of ciprofloxacin for mutated quinolone resistance-determining regions (QRDR) of target enzymes in SA1199B (Figure 9A). In addition, these results were obtained using a largely subtoxic concentration of compound **B**, which would also lead to a very reduced hemolytic effect of the α-hydrazido acid and thus to a larger therapeutic index.

On the contrary, compound **A** in a subtoxic concentration was able to potentiate the activity of ciprofloxacin only against SA1199B (ΣFIC = 0.75), albeit in this case a noticeable four-fold decrease in ciprofloxacin MIC in combination was obtained. The difference between the results of compounds **A** and **B**, especially the disappointing ΣFIC = 2 for the combination compound **A**/ciprofloxacin against SA1199, at the moment is not easily explainable. The synergy due to the increased uptake of quinolones and fluoroquinolones upon the permeabilizing action of antimicrobial peptides on bacterial membranes is a general phenomenon well documented in the literature for many bacteria [74,75], but for *S. aureus* additivity is much more common than synergy [67,71,76]. In the present case, further work is needed in order to draw conclusions about the more powerful synergy of compound **B** with ciprofloxacin. However, in the perspective of counteracting common bacterial resistance mechanisms, the potentiating effect of both compounds **A** and **B** for ciprofloxacin against the resistant strain SA1199B has to be considered valuable for future improvements and applications.

The indifferences showed by combinations of α-hydrazido acids with linezolid against LR-MRSa (ΣFIC = 1.03 for both compounds **A** and **B**), and with gentamicin against GR-CREc (ΣFIC values of 1.25 and 1.50 for compounds **A** and **B**, respectively, Table 2), can be explained in both cases with the lack of the necessary increased accumulation of first-line antibiotic when in combination with our amphiphilic compounds, even if the supposed mechanistic reasons are different.

Oxazolidinones, such as linezolid, inhibit protein synthesis by binding to the peptidyl transferase of the bacterial ribosome. When present, the *cfr* gene encodes an rRNA methyltransferase that catalyzes post-transcriptional methylation to the C8 position of nucleotide A2503 in 23S rRNA, causing a decreased binding affinity and leading to linezolid resistance [77]. In particular, the linezolid MIC rises from 1–4 μg/mL for *S. aureus* ATCC 29213 [55] to 16 μg/mL for *S. aureus* AOUC-0915 (LR-MRSa), then the MDR strain shows medium/low-level resistance (Figure 9B). Conversely, *aac(3)-IIa* gene, one of the most common resistance genes found in Gram-negative isolates, causes the covalent modification of gentamicin and other aminoglycosides, resulting in poor binding to the ribosome target and thus leading to resistance to these antibiotics (Figure 9C) [78]. For gentamicin, the resistant *E. coli* 288328 (GR-CREc) strain has a really low sensitivity to the antibiotic (MIC = 128 μg/mL), compared to the susceptible strain *E. coli* ATCC 25922 (MIC = 0.25–1 μg/mL) [55]. The best ΣFIC indices (1.03 for both compounds **A** and **B** toward LR-MRSa, 1.25 and 1.50 for **A** and **B**, respectively, toward GR-CREc) and their related single-drug concentrations (linezolid, 16 μg/mL and gentamicin, 128 μg/mL) indicate that the bacteriostatic activity of the combinations is mainly due to the conventional antibiotics, since no MIC difference between single drug and drug/α-hydrazido acids combination was observed (Table 2). It must be concluded that, for linezolid and gentamicin toward resistant *S. aureus* AOUC-0915 and *E. coli* 288328, respectively, the presence of amphiphilic permeabilizing compounds is not able to substantially increase their cytoplasmic concentrations.

A decreased uptake of linezolid has never been reported for *S. aureus*, while it was reported for an in vitro-selected linezolid-resistant *Staphylococcus epidermidis* mutant [79]. Therefore, the not so large increase in linezolid cytoplasmic concentration that would be necessary to counteract the low binding affinity to 23S rRNA, was not expected even in combination with α-hydrazido acids, in agreement with our experimental findings (Figure 9B). In the case of gentamicin, its polycationic structure is necessary to disrupt Mg^2+^ bridges between LPS molecules in the outer membrane, thus self-promoting the uptake. Mutations in LPS phosphates of *E. coli* were shown to decrease this self-promoted uptake and cause a large increase in gentamicin MIC [80]. In addition, the covalent modification of the drug triggered by the *aac(3)-IIa* gene, also present in GR-CREc [61,78], further enhances the overall resistance to gentamicin by strongly decreasing the binding to the ribosomal target. Thus, even though amphiphilic α-hydrazido acids were demonstrated to be able to favorably interact with the more positively charged LPS in the outer membrane of GR-CREc strain (Figure 3), leading to its permeabilization, their combinations with gentamicin were simply additive, because **A** and **B** at these low concentrations could not lead to the necessary 128/512-fold increase in cytoplasmic concentration of gentamicin that would tackle such a decreased affinity for the ribosomal target.

## 3. Materials and Methods

### 3.1. Density Functional Theory Calculations

The Density Functional Theory (DFT) calculations were performed with the Gaussian 16 Revision B.01 suite of programs [81], using the dispersion-corrected hybrid functional ωB97X-D3(0) [82] with the optimized parameters for the long-range Grimme’s dispersion correction with zero dumping [83], D3(0), and describing the solvent with the integral equation formalism-polarizable continuum model (IEF-PCM) method [84]. The internally stored 6-311G+(2d,p) basis set was used. The most stable conformation previously evaluated for an amphiphilic α-hydrazido acid hydrochloride having two linear octanoyl/octyl side chains on the N- and C-terminals [31] was used as a starting point for the construction and optimization of compounds **A** and **B** reported in Figure 1.

### 3.2. MD Simulations

A bacterial inner membrane model was generated using CHARMMGUI 3.8 [85] with 58 1-palmitoyl-2-oleoyl-*sn*-glycero-3-phosphoethanolamine (POPE) and 20 1-palmitoyl-2-oleoyl-*sn*-glycero-3-phosphoglycerol (POPG) lipid molecules per leaflet, following an already published model for the inner membrane composition of *E. coli* [86]. The lipid system was solvated by 6,156 transferable intermolecular potential with 3 points (TIP3P) [87] water molecules and 54 Na^+^ and 14 Cl^−^ ions to neutralize the net charge of the membrane and to reach the physiological conditions (0.15 M NaCl). The model with the reported composition was used to generate four different systems: a lipid system as it is, and other three in which 1, 2, and 3 molecules of compound **A** were added, respectively. The reason for the choice to include an increasing number of amphiphilic α-hydrazido acid molecules is related to the investigation of their different (possibly increasing) effect on the membrane permeabilization and also to highlight a potential aggregation trend of these molecules, so that they could locally induce a relevant damage onto the membrane itself. Each amphiphilic molecule was randomly placed in the water medium without any preferred orientation toward the membrane surface, with the aim to better reproduce the experimental conditions. All membrane systems had a simulation box of 6.82 × 6.82 × 8.5 nm, while one, two, and three chloride anions were added in the systems with one, two, and three amphiphilic molecules, respectively, as counterions. Molecular dynamics (MD) simulations have been performed using GROMACS 5.1.1 [88,89]. A minimization phase composed of 10,000 cycles of steepest descent algorithm followed by 5000 cycles of conjugate gradient minimization was used to converge to the energy threshold of 1000 kJ/mol/nm. Then, each lipid system was gradually equilibrated in their salt-aqueous environment through the six sequential equilibration steps, in which the reference temperature of 310 K was gradually reached. Atom velocities were then generated in the thermodynamic ensemble maintaining moles (N), volume (V), and temperature (T) (NVT) constant using the Maxwell distribution function with a generated random seed and a weak temperature coupling using the Berendsen thermostat. A time constant of 1 ps was applied to maintain the reference temperature (310 K) for the whole run. Verlet cutoff [90] was used in combination with Particle Mesh Ewald (PME) for electrostatics [91]. The cutoff for the calculation of the van der Waals force was set to 1.2 nm, while the force smoothly was switched to zero between 1.0 and 1.2 nm. After the equilibration steps, each system underwent 200 ns of MD simulation in NPT (moles, pressure, and temperature constant over the MD time) ensemble implementing an accurate leapfrog algorithm or interacting Newton’s equations of motion with a time step of 0.002 ps. The weak coupling was maintained also for pressure control (i.e., Berendsen barostat). For all simulation runs, the semi-isotropic conditions were set with a reference pressure of 1 atm and a time constant for coupling of 5 ps. A shift from the Nosé-Hoover [92] to the Parrinello-Rahman algorithm for pressure coupling [93] was operated for the production phase in NPT Ensemble. Different membrane parameters such as thickness, area per lipid, and deuterium order parameter were calculated to remark structural differences induced by the insertion of amphiphilic α-hydrazido acids. The analysis of the simulations’ trajectories was performed by means of VMD 1.9.3 [94] and CHIMERA 1.18 [95].

### 3.3. General Important Notes on the Use of α-Hydrazido Acid Hydrochlorides Solutions

As already reported, the hydrochlorides of α-hydrazido acids are surfactants, and their tendency to form foams can have a very negative impact on all the experiments in which a known and precise concentration is needed, and this is especially true for the most concentrated solutions [31]. In order to obtain consistent and reproducible results, withdrawal and addition times of at least three seconds were always used with Gilson pipettes, whereas the lack of bubbles was always visually checked whenever a 10 µL syringe was utilized. Vortexing the homogeneous stock solutions of α-hydrazido acids **A** and **B** before the experiments was also avoided. In addition, the mixing procedure within each well during the serial dilutions was always conducted carefully.

### 3.4. Determination of CMC

Critical Micellar Concentrations (CMC) were determined in 150 mM phosphate buffer solutions (PBS, pH 7.4) by using the fluorescent probe *N*-phenyl-1-naphthylamine (NPN), slightly modifying a previously described procedure [96]. Briefly, 6 μL of a 0.5 mM solution of NPN in acetone (final concentration, 1 μM) was added to 3 mL of PBS and stirred for 60 min at 37 °C. After determining the initial NPN fluorescence intensity, increasing concentrations of either compound **A** or **B** (1024 μg/mL, solutions in sterile water) were added. After 60 min of equilibration under stirring at 37 °C, the fluorescence was recorded, and data collected. All measurements were performed with a Perkin Elmer LS 50 spectrometer (Waltham, MA, USA), using quartz cuvettes with 10 mm path length, and operating with the following parameters: 350 nm (slit width 10 nm) for excitation and 420 nm (slit width 10 nm) for emission; photomultiplier voltage 600 V. The point of intersection between the two lines traced before (aqueous environment) and after the net raise in fluorescence (micellar environment) was used to calculate the CMC. Three tests were executed for each compound.

### 3.5. DNA-Amphiphiles Binding Assay

Gel retardation assay was performed as previously described with minor modifications [97]. Briefly, 100 ng of bacterial plasmid pBR322 (Sigma Aldrich, St. Louis, MO, USA) was mixed with different concentrations of either compound **A** or **B** in binding buffer (10 mM Tris buffer at pH 8.0 containing 5% glycerol, 50 μg/mL of bovine serum albumin, BSA, 1 mM ethylenediaminetetraacetic acid, EDTA, and 20 mM KCl). After incubation for 1 h at 37 °C, samples were run on 1% agarose gel electrophoresis in 1X TAE (Tris-acetate-EDTA) buffer. The DNA bands were detected by UV illuminator (Bio-Rad, Hercules, CA, USA).

### 3.6. LPS-Amphiphiles Binding Assay

The ability of amphiphiles to bind to LPS was determined using a fluorescent probe BODIPY-TR cadaverine (BC) (Sigma, St. Louis, MO, USA) displacement assay as previously described with minor modifications [98]. Briefly, PBS (150 mM, pH 7.2)-EDTA (1 mM) solutions of LPS from *E. coli* 0111:B4 (20 μg/mL) and BC (4 μg/mL) were mixed in a quartz cuvette containing 50 mM Tris buffer (pH 7.4) under stirring for 2 h at 37 °C. Appropriate amounts of aqueous solution of either compound **A** or **B** (1024 µg/mL) were then added to the mixture containing LPS:BC complex and left to equilibrate for 0.5 h under stirring at 37 °C. A solution containing LPS:BC complex and colistin sulphate (working concentration 60 μg/mL) was used as benchmark reference compound. Fluorescence intensity measurements were recorded (excitation λ = 580 nm, emission λ = 620 nm, slit width 10 nm in both cases) by using a Perkin Elmer LS 50 spectrometer. The absolute fluorescence values were converted to % ΔF as follows:

% ΔF (A.U.) = [(Ff − F0)/(F100 − F0)] × 100(1)

where Ff is the final fluorescence in the presence of a given concentration of added α-hydrazido acid, F0 is the initial fluorescence of LPS:BC complex, and F100 is the fluorescence upon addition of colistin (a prototype LPS binder) at a working concentration of 60 μg/mL, which was used as a positive control. Data are presented as mean ± S.D. (standard deviation). Statistical comparison of differences among groups of data was carried out using Student’s *t*-test. Values of *p* < 0.05 were considered statistically significant and values of *p* < 0.01 were considered highly significant.

### 3.7. Minimum Inhibitory Concentrations (MICs)

The MICs of α-hydrazido acid hydrochlorides **A** and **B** were determined according to the CLSI guidelines [99], using work solutions of both compounds in sterile water at a concentration of 1024 μg/mL. The bacterial strains included *S. aureus* ATCC 29213, AOUC-0915, SA1199, SA1199B (the latter two kindly provided by Kaatz G.W., Wayne State University School of Medicine, Detroit, MI, USA, and Sabatini S., University of Perugia, Perugia, Italy), and *E. coli* ATCC 25922 and 288328.

The strains were grown overnight in Brain Heart Infusion (BHI) broth and diluted in Mueller-Hinton II (MHII) broth (Oxoid S.p.a., Milan, Italy) to give a final concentration of 1 × 106 cfu/mL. Serial dilutions of the tested compounds in MHII broth (concentrations ranging from 256 to 0.25 μg/mL) were prepared in 96-well microtiter plate (Thermo Fisher Scientific, Waltham, MA, USA) (50 μL per well) and 50 μL of diluted bacterial suspension were added into each well. The wells with bacteria alone were used as positive growth control. Tetracycline (Merk Life Science S.r.l., Milano, Italy) was used as internal control, starting from a 1024 μg/mL working solution made from a stock solution at a concentration of 10 mg/mL. The plate was aerobically incubated at 37 °C for 24 h. All tests were performed in triplicate. The MICs were defined as the lowest concentrations of compounds inhibiting visible growth after 24 h of incubation.

### 3.8. Checkerboard Assays

The two α-hydrazido acid hydrochlorides (**A** and **B**) were tested, by checkerboard assays [55], in combination with tetracycline against *S. aureus* ATCC 29213 and *E. coli* ATCC 25922, with methicillin, linezolid and ciprofloxacin *S. aureus* against AOUC-0915, SA1199, SA1199B, respectively, and with colistin and gentamicin against *E. coli* 288328. The compounds were used as working solutions at a concentration of 64 μg/mL, to obtain by dilution a final concentration range of 16–0.25 μg/mL. Similarly, all antibiotics were used as working solutions corresponding to 64x MIC against each bacterial strain, to obtain by dilution a final concentration range corresponding to 16x-0.01x MIC.

Briefly, each well of the microtiter plate was inoculated with 50 μL of MHII broth. Then, 50 μL of working solution in water of the suitable α-hydrazido acid were added to each well of the first row and twofold serially diluted in the vertical direction. Subsequently, 50 μL of working solution in MHII broth of the suitable first-line antibiotic were added to each well of the last column and twofold serially diluted 1:2 leftward in the horizontal direction. Finally, 50 μL of bacterial suspension were added to every well of the plate, then the plates were incubated at 37 °C for 24 h. Each experiment was performed in triplicate. The fractional inhibitory concentrations (FICs) were calculated as the MIC of a drug in combination divided by the MIC of the same drug alone, and then the ΣFIC indices as the sum of FICs.

## 4. Conclusions

In this work, the amphiphilic α-hydrazido acid hydrochlorides, previously reported as structurally simple mimics of the action of antimicrobial peptides, were evaluated to describe their permeabilization mechanism of bacterial membranes and to clarify the synergistic effects when used with common antibiotics. First, the presence of a rapid permeabilizing action was demonstrated on two *E. coli* strains, showing that both the outer and inner membranes could be efficiently permeabilized. α-Hydrazido acids were also shown to interact strongly with LPS and disaggregate it.

The measured critical micellar concentrations were higher than MICs against all the bacteria tested, thus excluding the formation of aggregates. Then, the insertion and the gradual accumulation of α-hydrazido acids into the phospholipid bilayers were theoretically evaluated by means of molecular dynamics simulations, showing that the permeability of the membrane was increasingly affected by the addition of just one, two or three amphiphiles. A gel retardation assay confirmed that the action of these amphiphiles could be safely described, at least predominantly, as due to membrane(s) permeabilization.

The behavior of amphiphilic α-hydrazido acids when used with common antibiotics was ascertained against different sensitive and (multi)drug-resistant strains, using for these latter ones only the corresponding antibiotics to which they acquired resistance. Using compounds **A** and **B** in combination with tetracycline against sensitive *S. aureus* and *E. coli* strains showed that the bacterial membrane(s) permeabilizing action at low concentrations was effective in producing a much higher cytoplasmic concentration of tetracycline.

In the colistin-resistant *E. coli* 288328 (GR-CREc), the most likely explanation for the synergistic effect was that the remaining partial action of colistin and the effect on the same target of amphiphilic compounds could lead to an increase in membrane permeability.

In the case of methicillin/amphiphilic α-hydrazido acids combinations against *S. aureus* AOUC-0915 (LR-MRSa), it was impossible to resensitize the bacterium to such a high level to obtain ΣFICs within the most commonly accepted range for synergy, because the transpeptidase PBP2a had an exceedingly low sensitivity to methicillin. However, compound **A** at ½ MIC was still able to induce a remarkable ≥32-fold decrease in methicillin MIC, possibly due to some specific allosteric regulation by specific π-cation or π-stacking interaction(s) of the phenyl N-terminal group of compound **A** with some residue(s) of PBP2a.

The combinations of **A** or **B** with ciprofloxacin were evaluated against the susceptible *S. aureus* strain SA1199 and its resistant mutant SA1199B. In this case, compound **B** was synergistic with ciprofloxacin toward the resistant strain SA1199B, thus efficiently counteracting the action of NorA efflux pump and increasing the cytoplasmic level of antibiotic to such an extent that also overcame the low affinity of mutated QRDR in target enzymes.

The additivities found for both compounds **A** and **B** in combination with linezolid toward resistant LR-MRSa (*S. aureus* AOUC-0915) proved that the large effect of the post-transcriptional methylation in the ribosomal target 23S rRNA could not be surmounted by the increase in membrane permeability, even because linezolid is known to be able to easily enter into cytoplasm of staphylococci by itself.

Also in the case of the highly gentamicin-resistant *E. coli* 288328 (GR-CREc), the reason for the observed indifference for combinations with either compound **A** or **B** was the impossibility to reach the necessary huge increase in the cytoplasmic concentration of conventional antibiotic. Although subtoxic concentrations of the amphiphiles could permeabilize membranes, the polar and not amphiphilic nature of gentamicin made difficult a direct influx throughout an almost structurally intact outer membrane.

The obtained results provide valuable data and indications for the design of novel antibiotic adjuvants, able to restore the drug activity and to counteract the most common, yet troublesome, antibiotic resistance mechanism. Further studies devoted to the chemical modification and improvement of compounds **A** and **B**, especially taking into consideration di- and tricationic amphiphiles, are already ongoing in order to achieve an increased synergistic activity even with other antibiotics, as well as to evaluate their efficacy in in vivo infection models. Considering the frightening spread of antibiotic resistance and, conversely, the delay of the development of novel drugs, the use of extremely efficient antibiotic/adjuvant combinations seems a promising approach to be pursued in chemical, microbiological and medicinal research.

## Figures and Tables

**Table 2:** Lowest σfic indices for binary combinations of selected α-hydrazido acids and first-line antibiotics toward drug-sensitive and drug-resistant strains ^a^.

ΣFIC vs. Drug Sensitive Bacteria ([H]/[X]) ^b^ for Combinations with the Indicated Antibiotics					
Compd	S. aureus ATCC 29213 ^c^		S. aureus SA1199 ^d^	E. coli ATCC 25922 ^c^	
	Tetracycline		Ciprofloxacin	Tetracycline	
A	0.50 (1/0.0313)		2 (8/0.16)	0.38 (1/0.0625)	
B	0.38 (1/0.0156)		0.75 (4/0.08)	0.50 (2/0.125)	
ΣFIC vs. Drug Resistant Bacteria ([H]/[X]) ^b^ for Combinations with the Indicated Antibiotics					
	*S. aureus* AOUC-0915 (LR-MRSa) ^e^		*S. aureus* SA1199B ^f^	*E. coli* 288328 (GR-CREc) ^g^	
	Methicillin ^h^	Linezolid	Ciprofloxacin	Colistin	Gentamicin
A	0.5 < ΣFIC ≤ 0.53 (2/64)	1.03 (0.125/16)	0.75 (4/2.5)	0.31 (1/0.5)	1.25 (1/128)
B	0.5 < ΣFIC ≤ 1.0 (2/1024)	1.03 (0.125/16)	0.5 (4/2.5)	0.28 (2/0.25)	1.50 (4/128)

^a^ Cells with cases of synergy are reported in light green. ^b^ [H] indicates the concentration of α-hydrazido acid in μg/mL, whereas [X] are the concentrations of the indicated antibiotics in μg/mL. ^c^ MICs of tetracycline alone: 0.125 μg/mL for *S. aureus* ATCC 29213, 0.5 μg/mL for *E. coli* ATCC 25922. ^d^ Ciprofloxacin-susceptible *S. aureus* SA1199. MIC of ciprofloxacin alone: 0.16 μg/mL. ^e^ Linezolid- and methicillin-resistant *S. aureus* AOUC-0915. MICs of antibiotics alone: 16 μg/mL for linezolid, >1024 μg/mL for methicillin. ^f^ Ciprofloxacin-resistant *S. aureus* SA1199B. MIC of ciprofloxacin alone: 10 μg/mL. ^g^ Gentamicin- and colistin-resistant *E. coli* 288328. MICs: 128 μg/mL for gentamicin, 8 μg/mL for colistin. ^h^ The exact MIC for methicillin could not be obtained; thus, the upper and lower limits in reported ranges were calculated using the minimum possible value (2048 μg/mL) and an infinite value, respectively, for the actual methicillin MIC.

## Data Availability

The original contributions presented in the study are included in the article and Supplementary Materials; further inquiries should be directed to the corresponding authors.

## Supplementary Materials

The following supporting information can be downloaded at: https://www.mdpi.com/article/10.3390/molecules29174078/s1, Figure S1: Differently normalized variation of fluorescence intensity with time caused by different concentrations of compound **B** for the permeabilization of both outer and inner membranes in the *E. coli* ATCC 25922 and GR-CREc (*E. coli* 288328) strains; Figure S2: Normalized fluorescence intensity of a sample containing GR-CREc (*E. coli* 288328), 15 μM propidium iodide and a concentration of compound **B** equal to ¼ MIC; Figure S3: Normalized fluorescence intensity of a sample containing GR-CREc (*E. coli* 288328), 15 μM propidium iodide and a concentration of compound **B** equal to MIC; Figure S4: Computational analysis of membrane properties as a function of the number of molecules of compound **A** added; Figure S5: Mean square displacement of phospholipids in the presence of compounds **A** and **B**; Figure S6: MD snapshots of *α*-hydrazido acid **A** (stick) and membrane (van der Waals sphere); Figure S7: Representative structure of compound **A** in membrane; Table S1: Cartesian coordinates and energies of ωB97X-D3(0)/6-311+g(2d,p)/IEF-PCM(water) structures for compounds **A** and **B** as hydrochlorides.
