# Supplementary material for: Investigation on the Synergy between Membrane Permeabilizing Amphiphilic α-Hydrazido Acids and Commonly Used Antibiotics against Drug-Resistant Bacteria
Source: Molecules. 2024 Aug 28;29(17):4078. doi: 10.3390/molecules29174078 (PMC11397519; doi:10.3390/molecules29174078)
Supplement: Supplementary file 1 [file molecules-29-04078-s001.zip › molecules-3126256-supplementary.pdf]

Supplementary Material for

Investigation on the synergy between membrane permeabilizing amphiphilic α-hydrazido acids and commonly used antibiotics against drug-resistant bacteria

Cristina Minnelli [a,1], Gianmarco Mangiaterra [b,1], Emiliano Laudadio [a], Barbara Citterio [b,2,*], Samuele Rinaldi [a,2,*]

[a] *Department of Life and Environmental Sciences, Polytechnic University of Marche, Via Brecce Bianche, 60131 Ancona, Italy.*

[b] *Department of Biomolecular Science, Biotechnology Section, University of Urbino "Carlo Bo", Via Santa Chiara 27, 61032 Urbino, Italy.*

TABLE OF CONTENTS

Additional figures.. 3
*Differently normalized variation of fluorescence intensity with time caused by different concentrations of compound **B** for the permeabilization of both outer and inner membranes in the E. coli ATCC 25922 and GR-CREc (E. coli 288328) strains.. 3*
*Non-linear regression with the exponential rise to maximum kinetic scheme for the permeabilization of both outer and inner membranes caused by compound **B** at ¼ MIC against gentamicin and colistin-resistant E. coli 288328 (GR-CREc).. 5*
*Non-linear regression with the exponential rise to maximum kinetic scheme for the permeabilization of both outer and inner membranes caused by compound **B** at MIC against gentamicin and colistin-resistant E. coli 288328 (GR-CREc).. 6*

Cartesian coordinates and energies of ωB97X-D3(0)/6-311+g(2d,p)/IEF-PCM(water) structures for compounds A and B as hydrochlorides 9

Additional figures

*Differently normalized variation of fluorescence intensity with time caused by different concentrations of compound **B** for the permeabilization of both outer and inner membranes in the E. coli ATCC 25922 and GR-CREc (E. coli 288328) strains.*

The following graph allows to visually emphasize the non-promoted uptake of PI by the collection strain *E. coli* ATCC 25922 in the absence of added amphiphilic compound **B**, which was otherwise not easily distinguishable in the original Figure 2b in the main text.

To this end, the graph below was obtained exploiting the same data used for Figure 2b in the main text, but all the points belonging to a given strain (*i.e.*, either *E. coli* ATCC 25922 or GR-CREc (*E. coli* 288328) strain) were normalized taking as zero the initial fluorescence, and as value 100 the highest fluorescence obtained with the same strain when submitted to a concentration of compound B equal to its MIC. The following expression was used to calculate the new normalized values for fluorescence, y_{norm}, starting from the original values used for plotting Figure 2b, y_{orig}:

$$y_{norm} = ((y_{orig} - 100)/(y_{max} - 100))*100$$

where y_{max} is the highest fluorescence value measured for that given bacterial strain when compound **B** was used at its MIC.

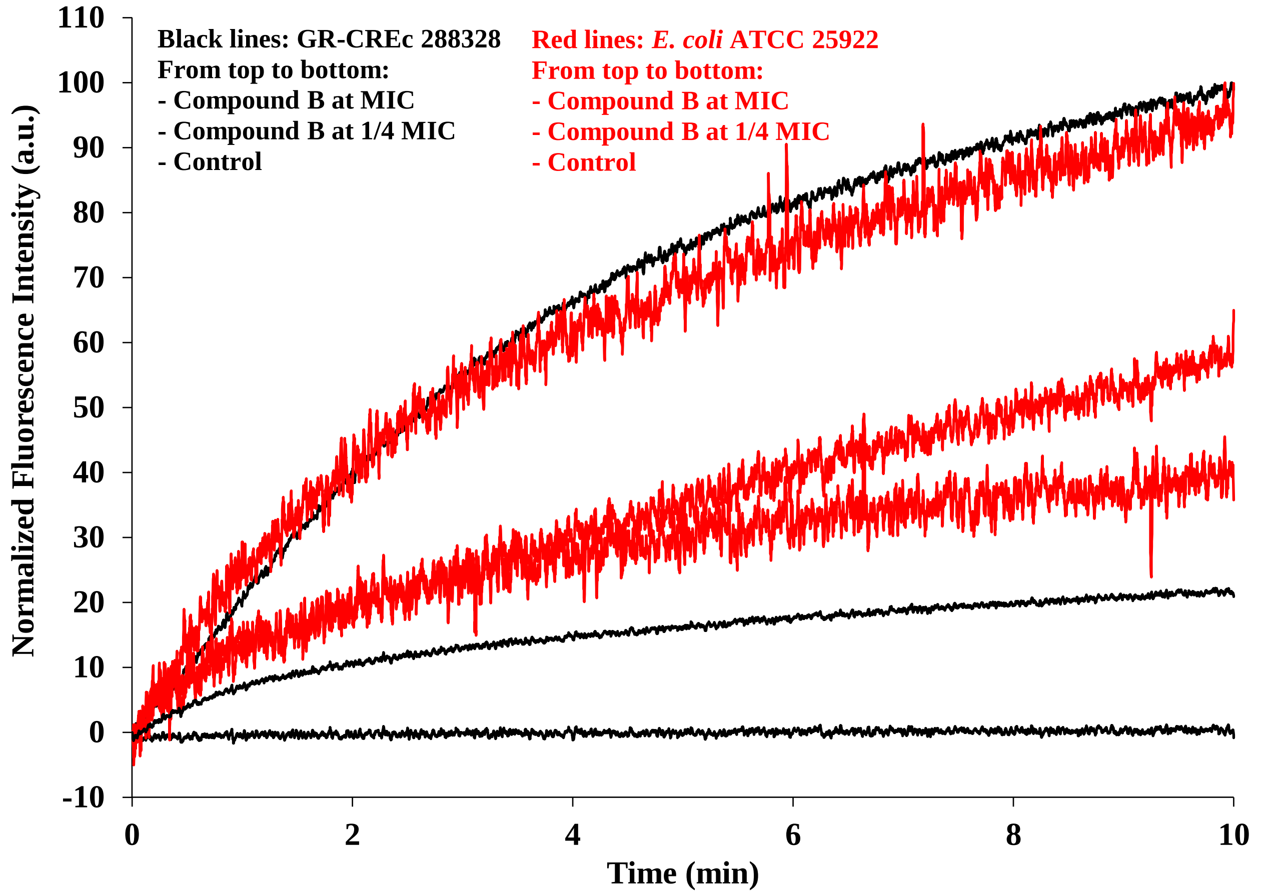

Figure S1. Variation of fluorescence intensity with time, normalized with the formula reported above, caused by different concentrations of compound **B** [control (compound **B** not added), ¼ MIC, MIC] for the permeabilization of both outer and inner membranes (fluorescent probe: PI) in the *E. coli* ATCC 25922 collection strain (red lines) and in the gentamicin and colistin resistant GR-CREc strain (*E. coli* 288328, black lines).

*Non-linear regression with the exponential rise to maximum kinetic scheme for the permeabilization of both outer and inner membranes caused by compound **B** at ¼ MIC against gentamicin and colistin-resistant E. coli 288328 (GR-CREc)*

Equation: Exponential Rise to Maximum; Single, 3 Parameter
F = F0+a*(1-exp(-k*t))

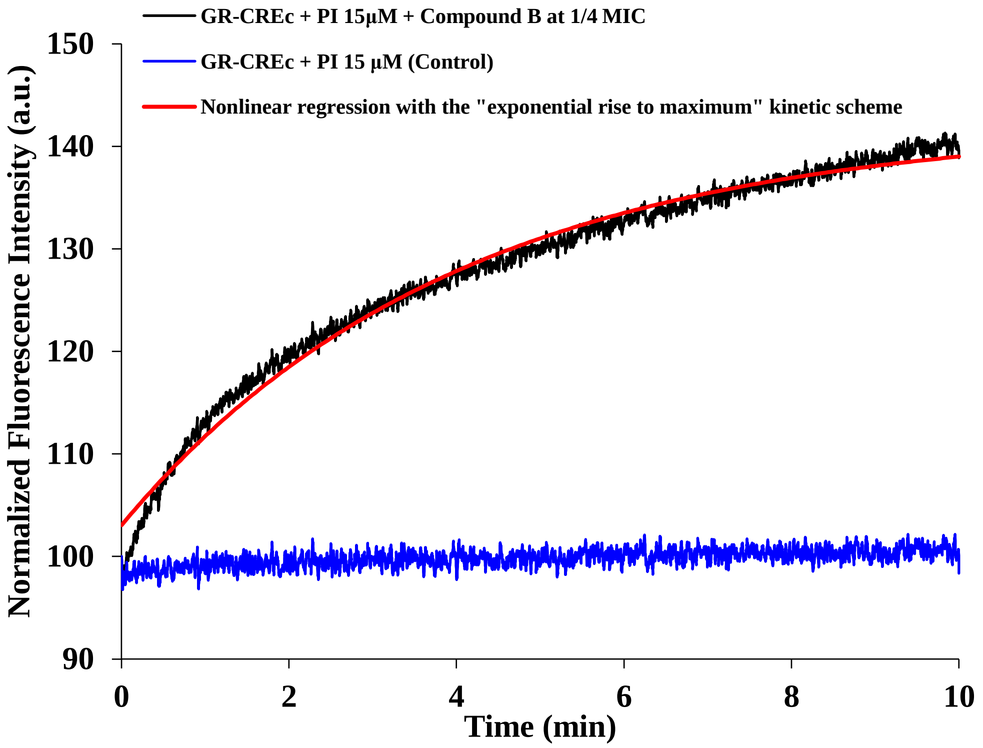

Figure S2. Normalized fluorescence intensity of a sample containing GR-CREc (*E. coli* 288328), 15 µM propidium iodide and a concentration of compound **B** equal to ¼ MIC.

*Non-linear regression with the exponential rise to maximum kinetic scheme for the permeabilization of both outer and inner membranes caused by compound **B** at MIC against gentamicin and colistin-resistant E. coli 288328 (GR-CREc)*

Equation: Exponential Rise to Maximum; Single, 3 Parameter
F = F0+a*(1-exp(-k*t))

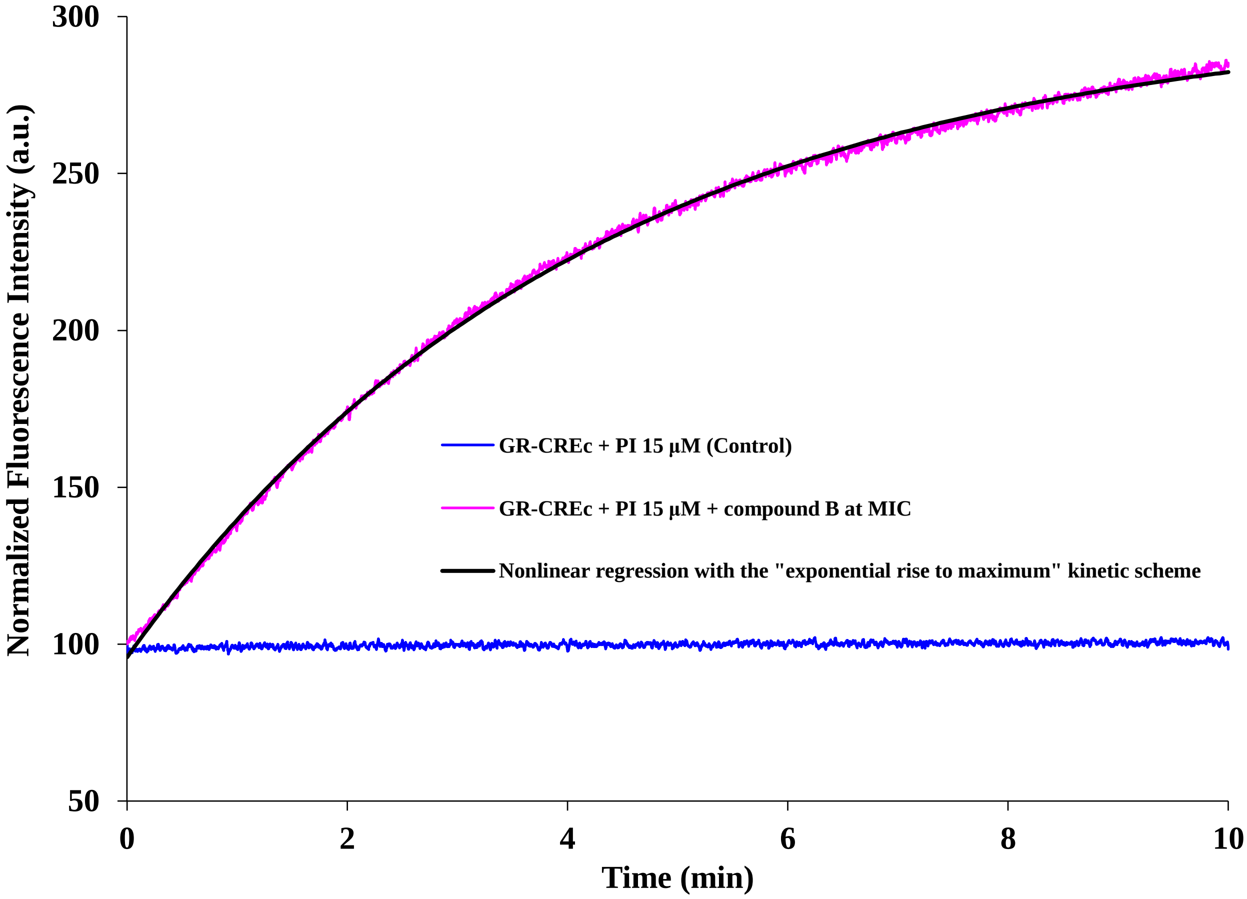

Figure S3. Normalized fluorescence intensity of a sample containing GR-CREc (*E. coli* 288328), 15 µM propidium iodide and a concentration of compound **B** equal to MIC.

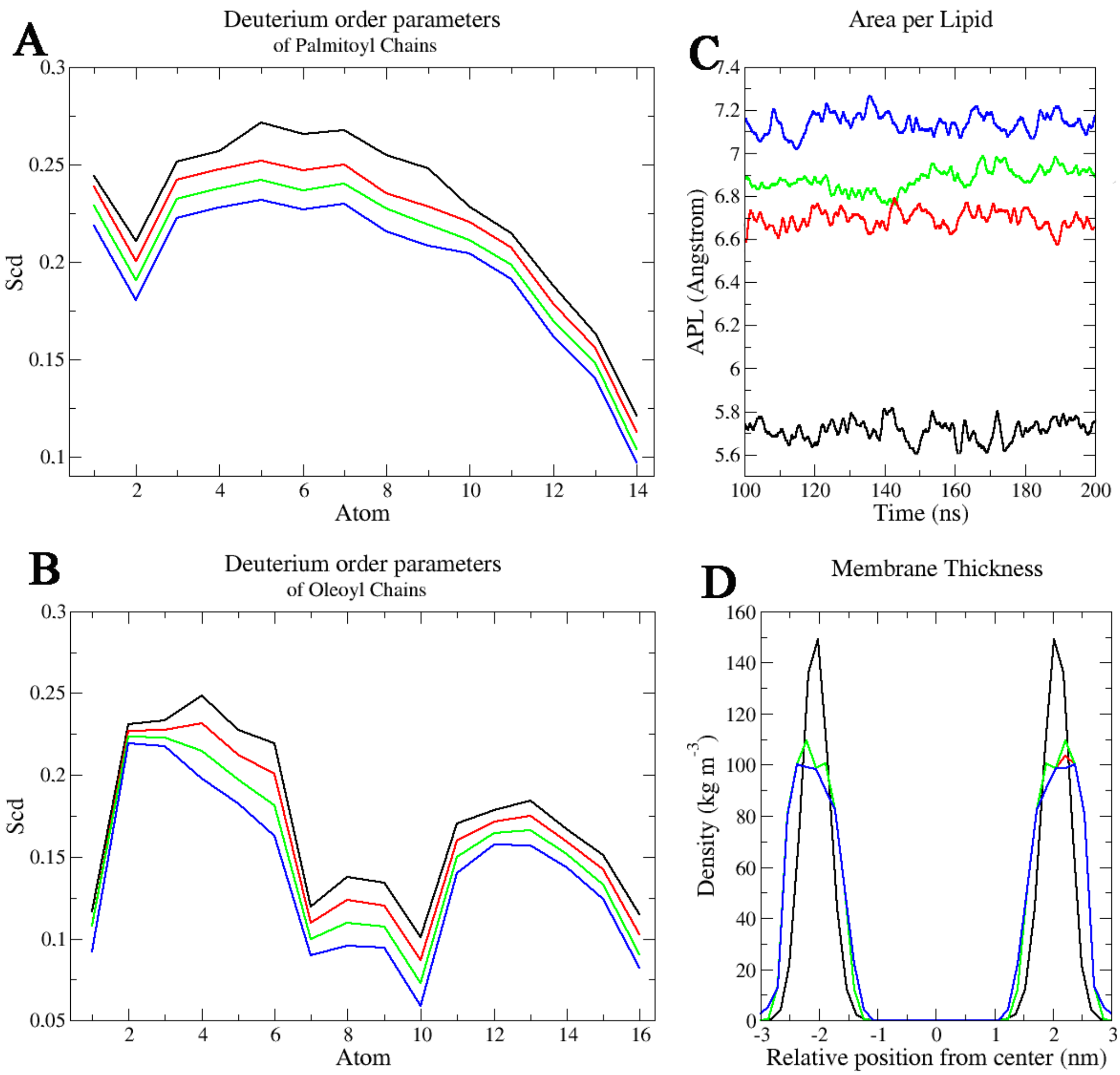

Figure S4. Computational analysis of membrane properties as a function of the number of molecules of compound **A** added (black lines: without compound **A**; red lines: one molecule of **A**; green lines: two molecules of **A**; blue lines: three molecules of **A**). (A) Deuterium order parameter of palmitoyl chains, (B) deuterium order parameter of oleoyl chains, (C) area per lipid, and (D) membrane thickness.

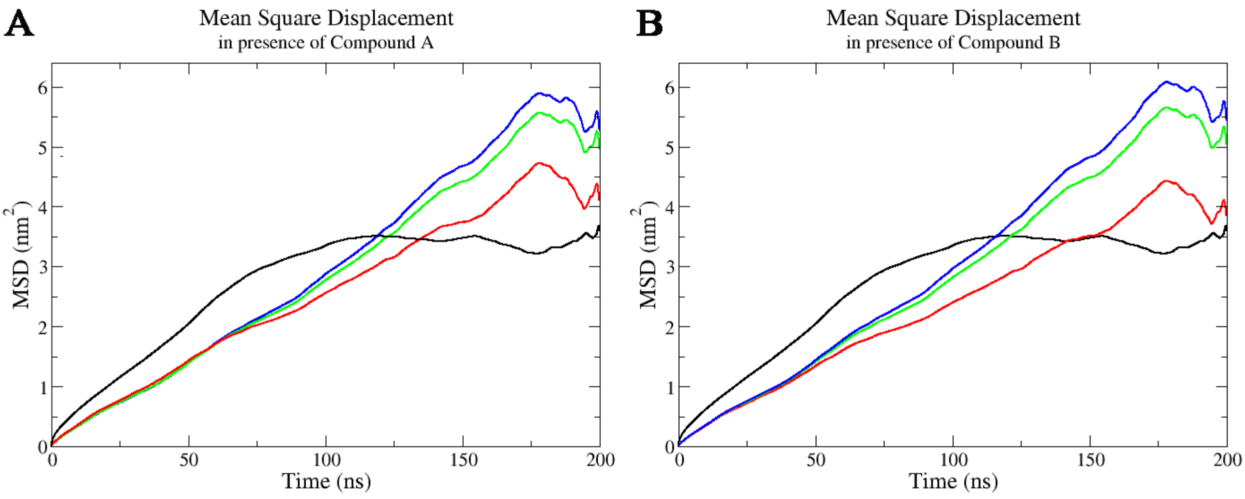

Figure S5. Mean square displacement of phospholipids in the presence of compounds **A** (a) and **B** (b). Black lines: without added compounds; red lines: one molecule added; green lines: two molecules added; blue lines: three molecules added.

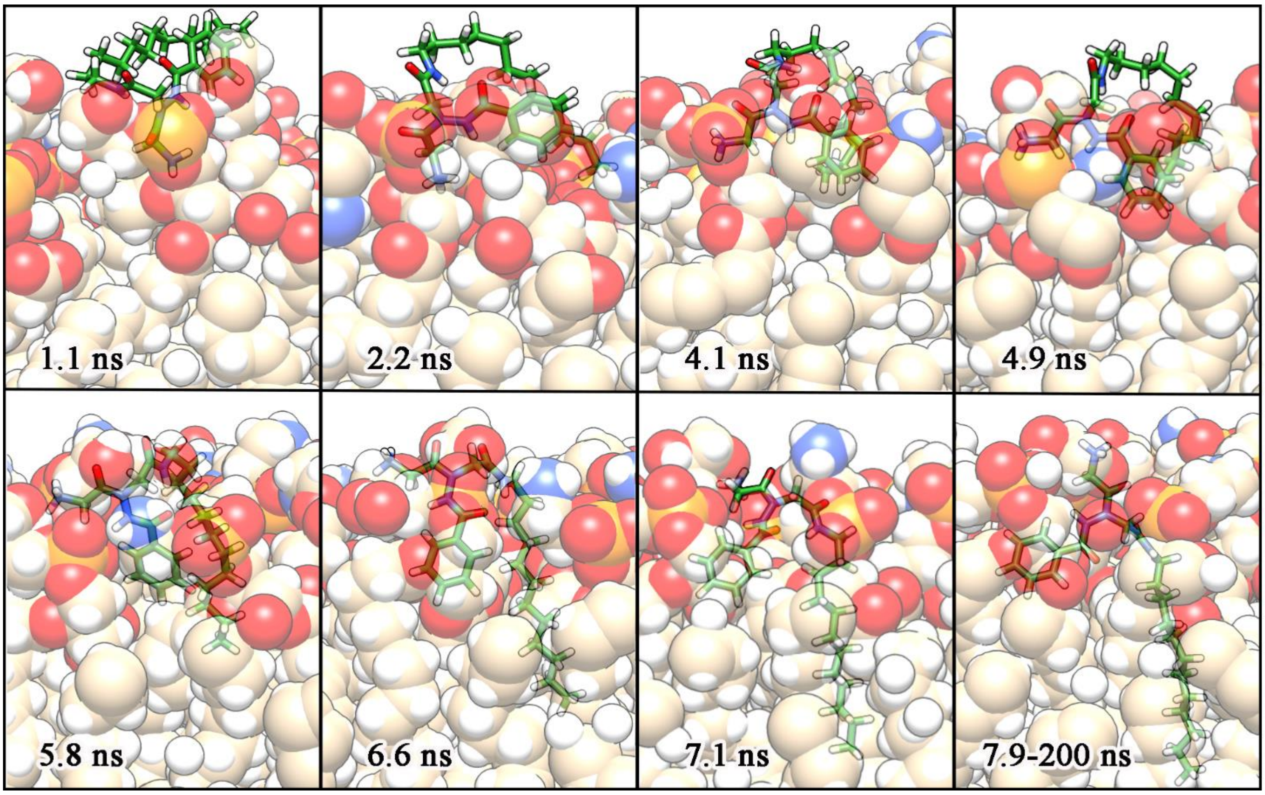

Figure S6. MD snapshots of α-hydrazido acid **A** (stick) and membrane (van der Waals sphere). O, N, P, and H atoms are highlighted in red, blue, orange, and white respectively. The C atoms of compound **A** are reported in green, while those of POPG and POPE are reported in light brown.

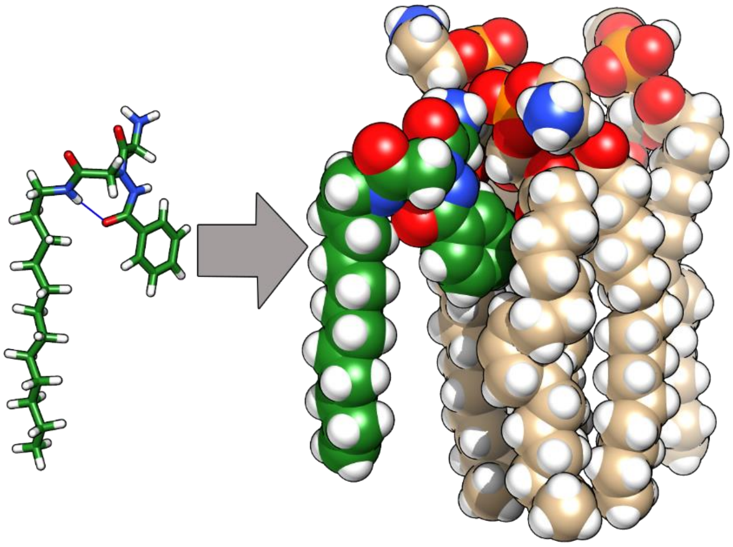

Figure S7. Representative structure of compound **A** in membrane. O, N, P, and H atoms are highlighted in red, blue, orange, and white respectively. The C atoms of compound **A** are reported in green, while those of lipids are reported in light brown. The blue line between the H and carbonyl O of compound **A** indicates the intramolecular H-bond.

Cartesian coordinates and energies of ωB97X-D3(0)/6-311+g(2d,p)/IEF-PCM(water) structures for compounds A and B as hydrochlorides

Compound A (Bz-HydrGly-NH$C_{12}H_{25}$ · HCl)

E = -1805.052369 au

C	-0.12376700	-0.15081600	0.88471800
O	0.15149300	-0.13704200	2.07370100
C	2.96257200	0.57281500	0.56639300
C	2.74373500	-1.76059100	-0.09634100
C	1.90927400	-2.96510900	0.32271600
O	1.78469300	-3.90834700	-0.44970400
N	0.84373800	-0.30078000	-0.07297700
O	4.17518400	0.48232100	0.63180100
H	0.73125700	0.07311900	-1.01513000
H	2.84316800	-1.79706500	-1.18373700
N	2.15598400	-0.49167000	0.31021400
H	3.73728800	-1.82933000	0.34646800

N	1.37367500	-2.93092500	1.55555700
H	1.41601400	-2.05072000	2.05315300
C	0.29504300	-3.83949600	1.93135200
H	0.49148600	-4.80592700	1.46539400
H	0.32105000	-3.97569300	3.01478300
C	2.31579700	1.92778000	0.81942300
H	1.28620900	2.00866100	0.48994700
H	2.38557900	2.14721900	1.88396200
H	4.11168700	2.77200800	0.23444200
H	2.92210500	2.75203300	-0.95179000
H	2.87041000	3.87524200	0.30279300
N	3.11461400	2.91685800	0.05927400
C	-1.04274700	-3.26920400	1.47958700
C	-2.26326600	-4.14273900	1.72172100
H	-0.97157300	-3.04561500	0.40808300
H	-1.19493300	-2.31201200	1.98729600
C	-3.51550600	-3.43311900	1.21601200
H	-2.14762000	-5.10773400	1.21296400
H	-2.36453100	-4.36037800	2.79236600
C	-4.81604700	-4.18993700	1.43890400
H	-3.58540400	-2.44905700	1.69807000
H	-3.39827600	-3.22611100	0.14447400
H	-4.76516800	-5.16734600	0.94184900
H	-4.94455000	-4.39411700	2.50981300
C	-6.02498600	-3.41874900	0.92150100
C	-7.34727900	-4.14506800	1.13034000
H	-5.88888200	-3.21092400	-0.14834200
H	-6.06542200	-2.43912600	1.41668500
C	-8.54848000	-3.36397000	0.61200900
H	-7.30732000	-5.12241000	0.63196400
H	-7.48294400	-4.35163800	2.19997300
H	-8.58743400	-2.38674000	1.11085100
H	-8.41143400	-3.15642700	-0.45730400
C	-9.87335800	-4.08756500	0.81849300
H	-10.00983600	-4.29567200	1.88774700

H	-9.83444900	-5.06434800	0.31888500
C	-11.07388900	-3.30513000	0.30099000
H	-10.93779600	-3.09630300	-0.76831500
H	-11.11359900	-2.32828700	0.80075300
C	-12.39979600	-4.02841100	0.50647000
H	-12.35833900	-5.00364900	0.00690200
H	-12.53392200	-4.23643600	1.57470200
C	-13.59126200	-3.23422400	-0.01561400
H	-13.49018300	-3.03937400	-1.08770400
H	-14.53191500	-3.76832000	0.14025600
H	-13.66713600	-2.26706000	0.49075300
C	-1.52446100	-0.05774400	0.37954300
C	-2.49378300	0.44322700	1.24448700
C	-1.89506900	-0.54191300	-0.87350000
C	-3.82446500	0.46484200	0.85883900
H	-2.19226500	0.79975900	2.22275900
C	-3.22857000	-0.52300800	-1.25505500
H	-1.15684300	-0.97449900	-1.54034400
C	-4.19279300	-0.02248100	-0.38990700
H	-4.57774100	0.85170200	1.53599800
H	-3.51674200	-0.91674100	-2.22317900
H	-5.23657500	-0.02128000	-0.68553900
Cl	1.97020900	1.50870500	-2.45042900

Compound B (Piv-HydrGly-NH$C_{12}H_{25}$ · HCl)

E = -1731.268882 au

C	0.10268900	0.21322600	0.46696500
O	0.02136800	-0.07399900	1.65191700
C	3.24008300	0.12400200	0.97254400
C	2.67623800	-1.98666800	-0.12341700
C	1.72015200	-3.09125400	0.30724700
O	1.70355900	-4.13775500	-0.33451000
N	1.21587600	-0.09027100	-0.26177200
O	4.33982300	-0.28031000	1.30387800
H	1.42215400	0.38178000	-1.13858300

H	2.71493400	-1.99342000	-1.21464800
N	2.32153600	-0.65720100	0.34225300
H	3.66992600	-2.22290500	0.25596800
N	0.98031200	-2.85771000	1.39873300
H	0.96074100	-1.91035800	1.75952600
C	-0.13113100	-3.71424000	1.78036100
H	0.07705900	-4.72094500	1.41529500
H	-0.17666600	-3.75563000	2.87186300
C	2.85772900	1.55674900	1.31920900
H	2.01198500	1.94755500	0.76588100
H	2.66214100	1.60846600	2.38953200
H	4.88588500	1.95546600	1.34894500
H	4.08783100	2.44159800	-0.05002000
H	3.95596200	3.33392100	1.37228100
N	4.03401200	2.39317200	0.98964000
C	-1.44090100	-3.18423600	1.21189500
C	-2.65376800	-4.01642100	1.59865200
H	-1.35369200	-3.13647500	0.11903600
H	-1.57726700	-2.15444300	1.55845900
C	-3.94929800	-3.42891800	1.05087500
H	-2.53218100	-5.04362900	1.23250200
H	-2.71867800	-4.08252300	2.69222800
C	-5.18724200	-4.22882300	1.43489100
H	-4.05680800	-2.39723100	1.41140000
H	-3.88254500	-3.36365600	-0.04305800
H	-5.08108600	-5.25942500	1.07219800
H	-5.25180800	-4.29347600	2.52882700
C	-6.47841100	-3.63208000	0.88870400
C	-7.72052300	-4.42392100	1.27802900
H	-6.41507500	-3.57111800	-0.20556500
H	-6.57986200	-2.59950100	1.24749700
C	-9.01107600	-3.82494200	0.73274500
H	-7.61983700	-5.45651600	0.91919900
H	-7.78317600	-4.48463400	2.37237200
H	-9.10968400	-2.79131800	1.08928600

H	-8.94955000	-3.76669800	-0.36179300
C	-10.25443900	-4.61317200	1.12571700
H	-10.31593500	-4.67079900	2.22031400
H	-10.15591300	-5.64697800	0.76968700
C	-11.54462400	-4.01386000	0.58021000
H	-11.48432000	-3.95719900	-0.51463600
H	-11.64300000	-2.97944800	0.93498900
C	-12.78944100	-4.80023500	0.97445400
H	-12.68942700	-5.83302600	0.61989200
H	-12.84811700	-4.85570300	2.06801000
C	-14.07175700	-4.18942300	0.42173700
H	-14.04511300	-4.15013000	-0.67156300
H	-14.95255000	-4.76635100	0.71454100
H	-14.20487800	-3.16626700	0.78643000
Cl	3.25516500	1.76331500	-1.93871600
C	-1.01571200	0.94890800	-0.27165700
C	-1.13865500	2.32875300	0.38914600
H	-1.32101400	2.23177500	1.46080400
H	-0.23174100	2.92325100	0.24041300
H	-1.97240500	2.87390900	-0.05982700
C	-2.30692200	0.15004700	-0.05859800
H	-2.51583800	0.02154600	1.00484700
H	-3.14256200	0.68493600	-0.51628600
H	-2.24037400	-0.83955300	-0.51929000
C	-0.75368700	1.11537300	-1.77016600
H	-0.63314200	0.15074200	-2.27027300
H	-1.60857700	1.62089000	-2.22424400
H	0.12964200	1.73082700	-1.97003300
